# Molecular cage-bridged plasmonic structures with well-defined nanogaps as well as the capability of reversible and selective guest trapping†
†Electronic supplementary information (ESI) available. CCDC 1492105. For ESI and crystallographic data in CIF or other electronic format see DOI: 10.1039/c7sc03536e


**DOI:** 10.1039/c7sc03536e

**Published:** 2017-11-20

**Authors:** Chen Wang, Li Tian, Wei Zhu, Shiqiang Wang, Ning Gao, Kang Zhou, Xianpeng Yin, Wanlin Zhang, Liang Zhao, Guangtao Li

**Affiliations:** a Department of Chemistry , Key Lab of Organic Optoelectronics & Molecular Engineering , Tsinghua University , Beijing 100084 , China . Email: LGT@mail.tsinghua.edu.cn; b Advanced Materials Laboratory , Sandia National Laboratories Albuquerque , New Mexico 87185 , USA

## Abstract

Creating well-defined plasmonic hotspots with enormous field enhancements as well as the capability of selectively trapping targeted molecules into hotspots is of critical importance and a prerequisite for numerous plasmon-assisted applications, but it represents a great challenge. In this work, a robust molecular cage decorated with thioether moieties at the periphery was designed and synthesized. By using the synthesized cage as a linker, a series of molecular cage-bridged plasmonic structures with well-defined nanogaps (hotspots) were fabricated in an efficient and controllable fashion. It was found both experimentally and theoretically that the nanogaps of about 1.2 nm created by the molecular cage in the resultant plasmonic structures led to a strong plasmon coupling, thus inducing great field enhancement inside the nanogaps. More importantly, the embedded molecular cages endowed the formed hotspots with the capability of selectively trapping targeted molecules, offering huge opportunities for many emergent applications. As a demonstration, the hotspots constructed were used as a unique nanoreactor, and under mild conditions two types of plasmon-driven chemical transformation were successfully performed. All the results clearly indicate that the integration of the host–guest chemistry of the molecular cage with the plasmon-coupling effect of metal particles afforded a new class of plasmonic structures, showing great potential for facilitating a broad variety of plasmon-based applications.

## Abstract 2

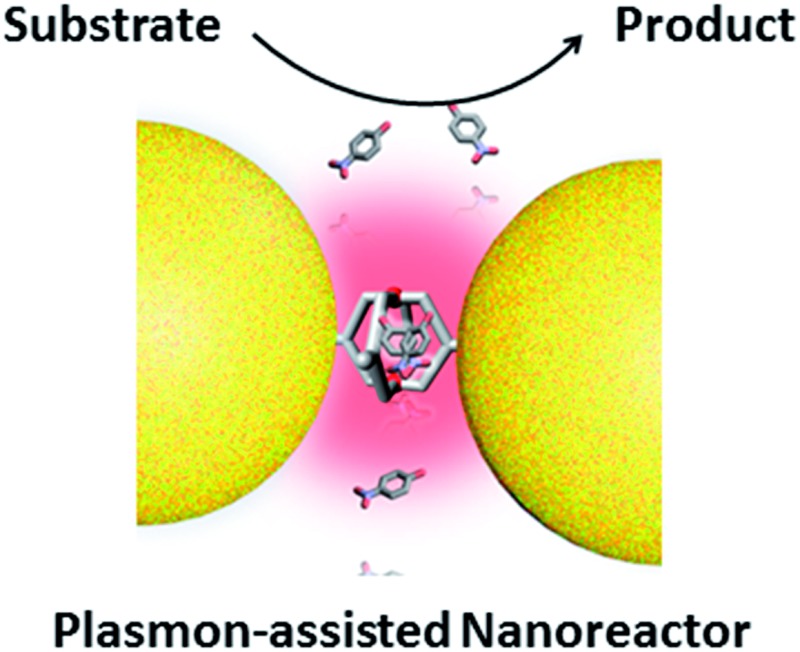
Molecular cage-bridged gold nanoclusters with well-defined hotspots were demonstrated as novel plasmon-assisted nanoreactors.

## Introduction

Noble metal nanoparticles (NPs) support unique localized surface plasmon resonances (LSPRs) through coherent oscillations of free electrons in metallic nanostructures under light illumination, and a strong electromagnetic field is induced in the close vicinity of the metal surface.^1^ Particularly, when such NPs are organized in closely spaced ensembles, a highly enhanced field (so-called hotspot) can be produced due to near-field plasmonic coupling of individual NPs at the nanometer gap junction between NPs.^1^,^2^ The resultant intense fields in hotspots make it possible to realize single molecule ultrasensitive detection *via* surface enhanced Raman scattering (SERS). In recent years, it was elucidated that the enormous field enhancement could also lead to the efficient generation of hot charge carriers,3*a* including energetic electrons and holes, which are powerful for driving chemical reactions and the conversion of optical energy into thermal or chemical energy.3*b* Thus, besides molecular sensing, the possibility to extract and use hot carriers generated offers tremendous opportunities and triggers new research in a broad variety of exciting applications such as photocatalysis and solar-to-chemical or electrical energy conversion.^4^ Apparently, for better and efficient implementation of these plasmon-assisted applications, creating hotspots with high field enhancements, especially those with an integrated capability of selectively trapping targeted molecules into hotspots, is of critical importance.

Generally, top-down and bottom-up strategies are employed for creating or engineering hotspot nanostructures.^5^ In the top-down strategy, traditional micro- and nanofabrication approaches such as lithography are employed to precisely and reproducibly manufacture plasmonic nanostructures. However, the current top-down fabrication techniques still face many challenges, including being time-consuming and cost-ineffective and in particular they have an intrinsic limitation of fabricating nanogaps of <10 nm.^5^ By contrast, the bottom-up self-assembly approach offers a practical and powerful alternative to create plasmonic structures with sub-10 nm gaps.^5^ Particularly, controlled assembly using colloidal chemistry, which is among the most promising bottom-up approaches, is an emerging and promising method for high-yield production of plasmonic nanoclusters with small interparticle gaps. Over the past 20 years, various colloidal methods have been developed for engineering hotspots.^5^ In pioneering work by Feldheim *et al.*, organic thiol-linkers were used to direct the organization of NPs.^6^ Alivisatos, Nam and Mirkin *et al.* have prepared well-organized NP ensembles with complex and hierarchical nanostructures *via* DNA.^7^ Although great progress has been made in the engineering of hotspots, many challenges still remain regarding the further development of this field.^5^–^7^ The utility of linking elements (for example, organic molecules, nucleic acids, peptides, DNA, polymers, and proteins) affords well-defined plasmonic nanostructures, but often passivates the plasmonic surface and thereby prevents the targeted guests from entering the hotspots.^5^ For the case of molecules that have low molecular affinities for a metal surface, the efficient capture of such species around the metal surface still represents a serious problem.^5^ Until now, the highly desired dense hotspot plasmonic systems with great field enhancement and with the capability of selectively placing targeted molecules into hotspots are rather limited.^5^

**Fig. 1:**
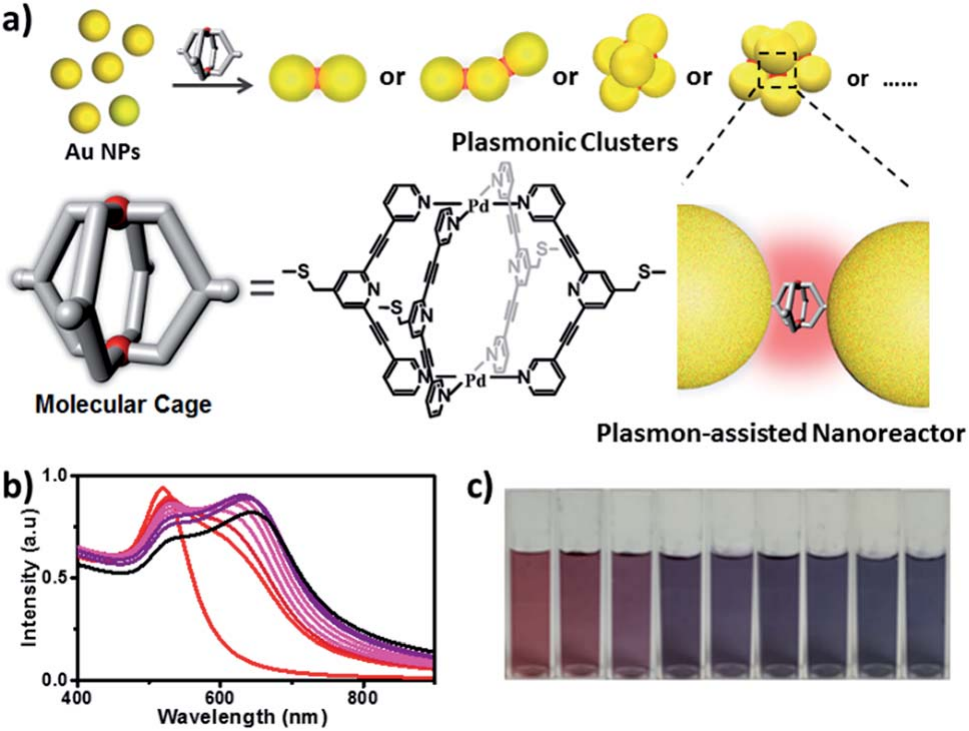
(a) Schematic illustration of the preparation of cage-bridged clusters with a well-defined nanogap; (b and c) UV-vis spectra and evolution of the color of the Au NP solution upon addition of the molecular cage.

In this work, we describe a new M_2_L_4_ metal–organic molecular cage decorated with thioether moieties at the periphery (Fig. 1a). We found through well-known S–Au chemistry that such a molecular cage could assemble gold NPs in microfluidic droplets to efficiently afford a series of molecular cage-bridged gold nanoclusters with well-defined nanogaps in a controllable fashion, including dimers, trimers, and tetramers, or clusters with large coordination numbers (CNs). Moreover, by utilizing a layer-by-layer and a template-guided approach three-dimensional (3D) dense hotspot substrates and an ordered cluster array system were also fabricated, respectively. The nanogaps created by the molecular cage in the resultant plasmonic structures are 1.2 nm, which is consistent with the cage size. Experimental results and theoretical simulation showed that the small nanogaps in these structures result in strong plasmon coupling, thus inducing great field enhancement inside the nanogaps. More importantly, it is found that the molecular cages located in hotspots exhibit sufficient stability and retain their host–guest chemistry, enabling hotspots to have the capability of selectively trapping guest molecules. Thereby, the integration of the host–guest chemistry of the molecular cage with the plasmon-coupling effect of metal nanoparticles affords a new class of plasmonic structure, offering tremendous opportunities for emergent plasmon-based applications. As a demonstration, hydroquinone (HQ) and cisplatin (CP), which have low molecular affinities for gold surfaces, were selectively detected with a limit of detection down to 10^–8^ and 10^–10^ M using the created hotspots as the SERS substrate. Furthermore, the created hotspots were also used as a unique nanoreactor to facilitate the plasmon-driven chemical conversions. The photoreduction of 4-nitrophenol (4-NP) under mild conditions as well as the cycloaddition from 4-ethynylphenol (4-EP) and 4-azido-phenol (4-AP) without catalysts were successfully realized. All the results indicate that the molecular cage-bridged plasmonic clusters described are new plasmonic nanostructures with advanced functionalities.

## Results and discussion

The organic ligand **L** [3,3′-(4-((methylthio)methyl)pyridine-2,6-diyl)bis(ethyne-2,1-diyl)dipyridine] was synthesized from 2,6-dibromoisonicotinic acid (Scheme S1†) to create the cage used. Through vapor diffusion of diethyl ether into the acetonitrile solution of ligand **L** (2 equiv.) and [Pd(CH_3_CN)_4_](X)_2_ (1 equiv.) at room temperature (X= BF_4_^–^ or OTf^–^), the colorless cage single crystal was achieved after three days with good yields of 79–93% (see details in ESI†). The cage structure was confirmed by single-crystal X-ray analysis. Each palladium is bridged by four ligands **L**, resulting in a lantern cage that contains an open and accessible internal cavity. The Pd–Pd distance is 11.8 Å, and the average distance between two facing pyridyl rings is 10.7 Å (Fig. S1a and b and Table S1†). The crystals are dissoluble in acetonitrile to form stable isolated molecular cages. Compared to the free ligand, due to the electron withdrawing effect of Pd^2+^ all the protons of the formed cage are shifted downfield (Fig. S1c†). Diffusion-ordered NMR (DOSY) measurements also showed that the formation of the cage causes much slower diffusion (*D* = 4.9 × 10^–10^ m^2^ s^–1^) than the free ligand **L** (*D* = 1.9 × 10^–9^ m^2^ s^–1^), which is indicative of the formation of larger chemical species (Fig. S2†). Furthermore, ESI-MS analysis confirms the Pd_2_L_4_ composition of the formed molecular cage with a series of prominent peaks for [Pd_2_L_4_(BF_4_)_(4–*n*)_]^*n*+^ (*n* = 2–4) (Fig. S1d†).

The host–guest behavior of the synthesized molecular cage was studied using five guest molecules with different size, shape and charge, including CP, 4-NP, HQ, *cis*-dichlorobis-(triphenylphosphine) platinum(ii) (DCTP) and 1,4-phenyldiamine hydrochloride (PDA) using ^1^H NMR, UV-Vis and ESI-MS (Scheme S2 and Table S2†). In our work, the Pd_2_L_4_ molecular cage used is soluble in CH_3_CN, and thus all of the cage’s host–guest chemistry was examined in CH_3_CN. The encapsulation behavior of the guest CP in the cage was first studied. In this case, due to the insolubility of cisplatin in CH_3_CN, performing a UV-Vis as well as a NMR titration experiment between the cage and cisplatin is impossible. Thus, ^1^H NMR and ESI-MS were employed to analyze the solution obtained from the sonication (10 min) of the suspension of CP in the CD_3_CN solution of the cage. As shown in Fig. S3,† in comparison with the NMR spectrum of the pure cage solution, a discernible downfield shift (Δ*δ* = 0.06 ppm) and a broadening of the internal proton H_a_ of the cage were detected in the NMR spectrum of the mixed solution, indicating that the guest CP was trapped in the molecular cage. Indeed, the ESI-MS result (Fig. S4†) shows the formation of a [Pd_2_L_4_@(cisplatin)_2_]^4+^ complex with an intense peak at *m*/*z* = 544.28, confirming that two cisplatin molecules were trapped in the cage. In fact, the same encapsulation behavior of the CP was also observed in a similar molecular cage only without the thioether moieties at the periphery.^8^

For the case of guest HQ and 4-NP, the trapping ability of the Pd_2_L_4_ cage was examined using ^1^H NMR, ESI-MS and UV-Vis methods. Due to the formation of hydrogen bonding between the guest and the pyridine moieties of the cage, the protons (H_a_ and H_b_) of the cage were most shifted upon HQ and 4-NP encapsulation (Fig. S5 and S9†), which is indicative of a strong binding affinity. Based on the ^1^H NMR titration experiments, it was found from the Job plots (Fig. S6 and S10†) that one HQ and two 4-NP molecules were trapped in one Pd_2_L_4_ cage, respectively. Through the UV-vis titrations (Fig. S7 and S11–S13†) the association constants of 1.155 × 10^6^ M^–1^ and 1.19 × 10^4^ M^–1^ were found for the case of HQ and the case of 4-NP, respectively. The ESI measurements further confirmed the results of the NMR titrations. The formation of the host–guest complex [Pd_2_L_4_@HQ]^4+^ with a major peak at *m*/*z* = 437.8 was found for the case of HQ (Fig. S8†). Similarly, the formation of the host–guest complex [Pd_2_L_4_(BF_4_)@(4-NP)_2_]^3+^ with the peak at *m*/*z* = 648.8 in the ESI-MS spectrum was also detected for the case of 4-NP (Fig. S14†). In our case, some mass spectra of the host–guest complexes displayed a series of [Pd_2_L_4_(BF_4_)_*n*_@guest]^(4–*n*)+^ (*n* = 1–3) peaks along with the peaks due to fragmentation of the cage under a certain spray voltage. In fact, a similar phenomenon was also observed in the literature.8*c* After the optimization of the experimental conditions, including the spray voltage, capillary temperature and ion accumulation time, the mass spectra actually supported the formation of the host–guest complexes, and the assignments for the observed peaks in the ESI spectra are also given in Fig. S4, S8 and S14.† In our work, we attempted to obtain the corresponding single crystals of the host–guest complexes with the expectation of gaining insight into the exact nature of host–guest interactions at the molecular level, but unfortunately failed. In addition, DOSY experiments of host–guest complexes were performed. Each of the guest and host proton signals in the individual spectra show the same diffusion coefficients (Fig. S15–S17†), clearly indicating that the guest used is inside the cage host.

For the case of guest DCTP (Table S2†), due to its large molecular size, the trapping of DCTP within the molecular cage was not detected using NMR spectroscopy (Fig. S18†). Probably due to the charge repulsion, the encapsulation of the positively charged guest PDA in the positively charged cage was also not observed (Fig. S18†). The ESI-MS measurements further confirmed the observed NMR results. As shown in Fig. S19–S20,† no corresponding mass peaks of host–guest complexes could be observed for both cases of DCTP and PDA, and only the peaks of the cage [Pd_2_L_4_(BF_4_)_(4–*n*)_]^*n*+^ and the ligand **L** were found. This considerable binding difference indicates the capability of the Pd_2_L_4_ cage for selective guest capture. It should be noted that due to the limited solubility of DCTP and PDA in the CH_3_CN solvent, the UV-Vis and NMR titrations between the cage and guests cannot be performed.

The assembly of Au NPs with the aid of the Pd_2_L_4_ cage was firstly tested in a bulk gold colloidal solution and monitored with a UV/Vis spectrometer. The Au NPs with a diameter of *ca.* 15 nm, prepared by the reduction of HAuCl_4_ with sodium citrate, employed in our case, initially show typical localized surface plasmon resonance (LSPR) absorption at 525 nm. As shown in Fig. 1b, upon the addition of a 2 μL droplet of a DMSO solution of the cage into the colloidal Au NPs, the LSPR absorption shifts gradually from 525 nm to 646 nm, and the color of the colloidal solution changes accordingly from wine red to dark blue (Fig. 1c). These results clearly indicate that the aggregation of Au NPs occurred. Indeed, huge aggregated Au NPs were detected under TEM observation (Fig. S21†). Based on this preliminary result, in our work, to achieve well-defined cluster structures, the controlled assembly of Au NPs and the molecular cage was performed in discrete microdroplets in a microfluidic device (Fig. S22†). Briefly, both solutions of Au NPs and the molecular cage were simultaneously pumped through separated and individual hemi-spherical orifices into a microchannel to merge and form monodisperse droplets in a continuous oil phase. The fusion of Au NPs and cage molecules in microdroplets triggered the self-assembling process, affording the well-defined cage-bridged plasmonic clusters. It was found that by adjusting the preparation conditions such as the concentration of Au NPs or the cage, the ratio of both components as well as the size of the droplets, the assembling that occurred could be precisely controlled, and a series of plasmonic nanoclusters is accessible in an efficient fashion after simple purification by density gradient centrifugation (Fig. S23†). It should be noted that the choice of thioether instead of a thiol group for decorating the molecular cage is critical for the related assembling process due to its relatively slower kinetic interaction with Au.

**Fig. 2:**
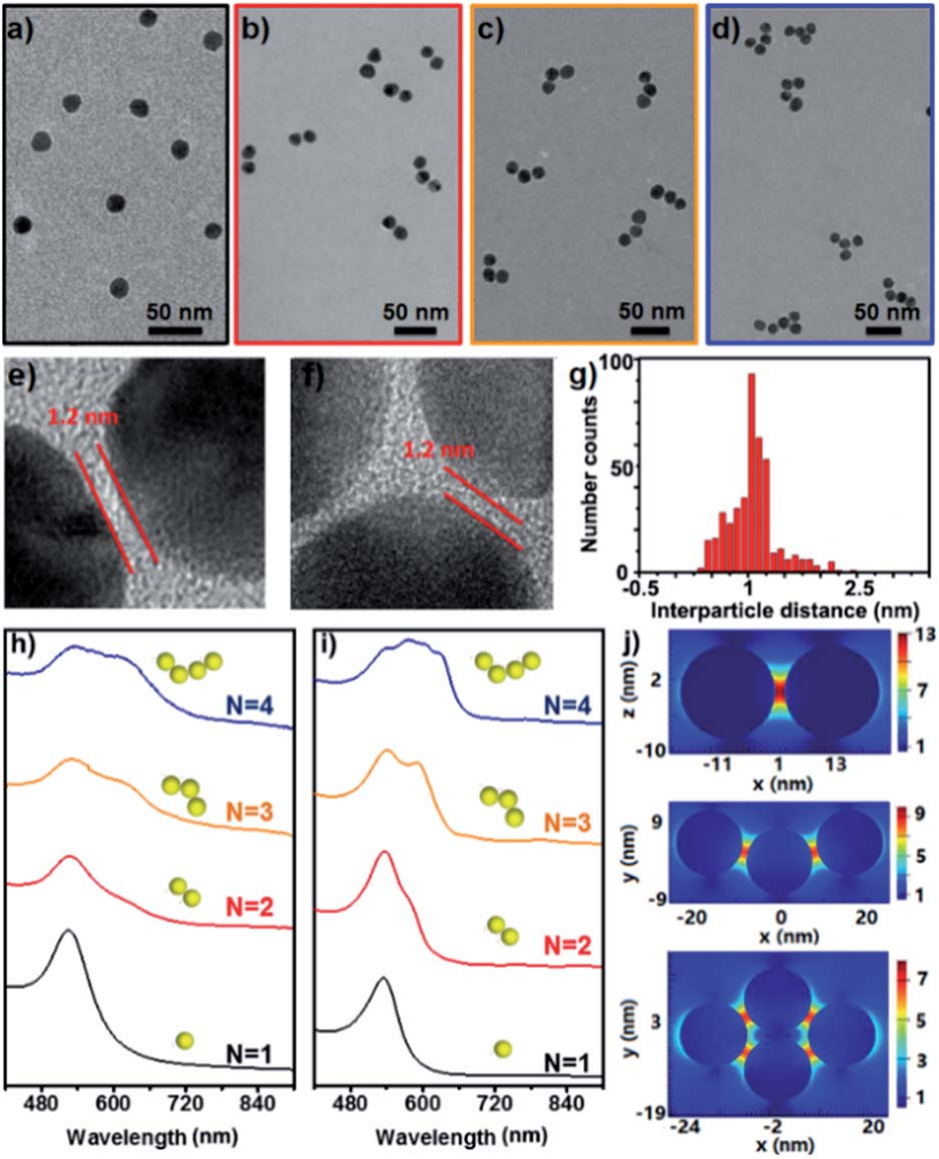
TEM images of the starting Au NPs (a), the resultant clusters (b–d) and HR-TEM images of the dimers (e) and trimers (f) with nanogaps; histogram of the gap distance in the formed clusters (g); experimental (h) and simulated (i) UV/vis spectra of clusters with CNs from 1 to 4; simulated distribution of the electric field intensity around Au NPs at 633 nm (j).

Fig. 2a–d show the representative TEM images of the starting Au NPs and the prepared simple Au clusters including dimers, trimers and tetramers. The narrow gap between two NPs in the clusters was measured to be *ca.* 1.2 nm (Fig. 2e–f), which was in agreement with the geometrical size of the cage used. Also an average longitudinal length of 48 nm was measured for the linear trimer; this length was quite well-matched with the geometrical prediction for the three 15 nm nanoparticles with two interparticle distances of 1.2 nm. An extensive collection of dimers and trimers was investigated with HR-TEM, and the histogram of gap distances between the closest neighbours indicates the narrow distribution of the small gap (Fig. 2g). The successful synthesis of different populations of clusters offers the opportunity to study the structure-dependent optical properties. Fig. 2h–i show the measured and calculated UV/vis spectra of the Au clusters with CNs ranging from 1 to 4. Clearly, the monomer was dominated by a single absorption band at 525 nm, while the optical response of the dimers, trimers, and tetramers was reminiscent of the elongated anisotropic NPs, characterized by two or more complex bands attributed to the transversal and longitudinal plasmonic modes. The good and reasonable agreement of the experimental spectra with the calculated counterparts reveals an efficient near-field coupling of plasmon modes through the cage-bridged small gaps (Fig. 2j), corroborating the optical behavior expected from plasmon hybridization.

**Fig. 3:**
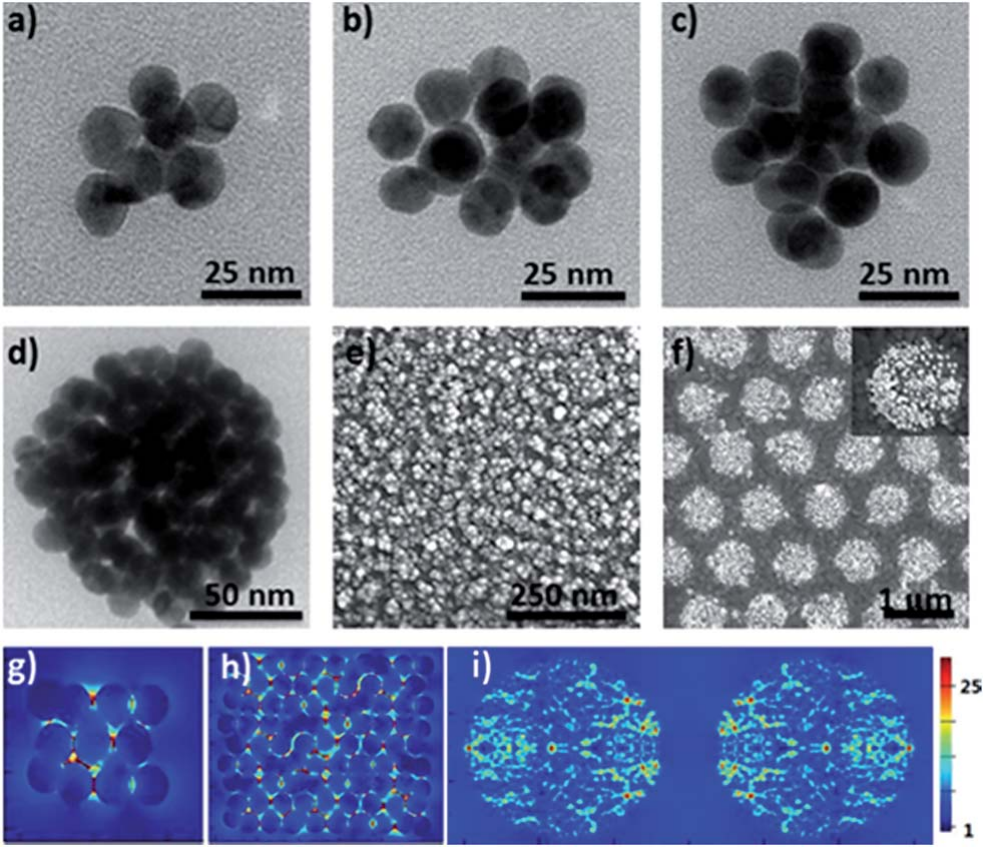
TEM images of plasmonic clusters with higher CNs (a–d); SEM image of the plasmonic substrate (e) prepared with the layer-by-layer method and cluster array (f) with high CNs; simulated distribution of the electric field intensity for the clusters, the plasmonic substrate and the cluster array at 633 nm (g–i).

Furthermore, the efficient synthesis of nanoclusters with high CNs is also possible by changing the preparation conditions such as the concentration ratio of Au NPs and the cage, as shown in Fig. 3a–d. Compared to simple clusters, these large symmetric ones (so-called 3D hotspot architectures) possess a higher density of hotspots and improved utility of hotspots in all three dimensions, and at the same time achieve higher tolerance in focus misalignment under external light illumination (Fig. 3g).5*b* In agreement with the results in the literature,^9^ it was also found that with the increase of CNs the SERS enhancement factor (EF) of clusters dramatically increased and could reach up to 10^6^ orders of magnitude (Fig. S25†). The enhancement is associated with the gaps because the number of gaps increases strongly with the CN. This result was in agreement with that reported in the literature,^9^ namely, that the EF is proportional to the number of gaps in plasmonic clusters. Moreover, by exploiting the layer-by-layer method the synthesized molecular cage could be used as a glue to construct large surface plasmonic nanostructures with dense 3D hotspots (Fig. 3e and h), which are highly desired for SERS applications.5*b* In particular, when a template was employed in the assembling process, hierarchical cluster arrays could also be facilely generated (Fig. 3f and S24†), and both the near-field and far-field plasmonic couplings result in a stronger local field enhancement in the plasmonic substrates (Fig. 3i).^10^

The successful incorporation of the Pd_2_L_4_ cage into the formed Au clusters or substrates was also confirmed by Raman and XPS studies. Compared to the Raman spectrum of the Pd_2_L_4_ cage, the typical bands of the cages appear in the SERS of the prepared clusters or substrates (Fig. S26†). And XPS spectra reveal the existence of Au4f (83.96 eV) as well as S2p (168.2 eV), Pd3d (334.94 eV) and N1s (399.3 eV), and especially the C≡C, C=C and C=N bands (Fig. S27†). These results indicate that the cages integrated into the Au clusters remain intact. More importantly, we found that the cages localized in the hotspots demonstrated sufficient stability in different media, including DMSO, C_2_H_5_OH, CH_2_Cl_2_ and THF, and after continuous illumination for 2 h the SERS spectra of the cage-bridged Au clusters remain unchanged, which is indicative of the good stability of the cages embedded in the plasmonic substrate. However, when treated with HCl (pH = 3) or NaOH solution (pH = 10), the fabricated substrate structure gradually diminished, which was indicative of the instability of the embedded cages in acidic and alkaline solutions (Fig. S28 and S29†).

**Fig. 4:**
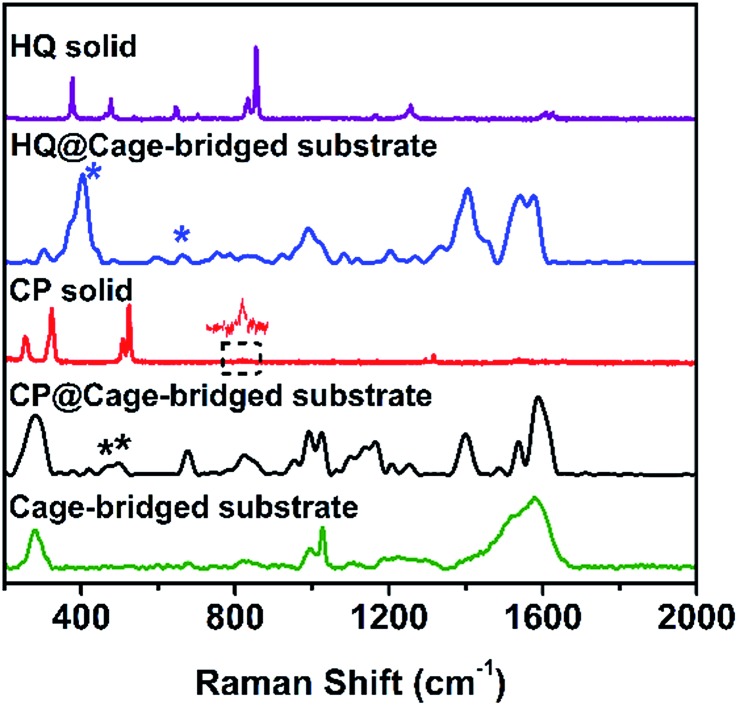
From top to bottom: Raman spectra of HQ, HQ trapped in the cage-bridged substrate, CP, CP trapped in the cage-bridged substrate, and the cage-bridged substrate.

To address the key issue that the molecular cages embedded in the Au clusters still retain their host–guest property, the plasmonic substrates described above were employed in related tests. Concretely, the substrates were immersed into a DMSO solution of different guest molecules (10^–4^ M) for a given time (2 h), and after being thoroughly washed with DMSO and ethanol the substrates were checked using the SERS technique. Fig. 4 displays the collected SERS spectra after the treatment of the plasmonic substrates with different guest molecules. As expected, the trapping of the small guest CP in the hotspots was detected, and the characteristic peaks of CP at 483 and 501 cm^–1^ (asymmetric and symmetric stretching doublet vibrations *ν*Pt-NH_3_) appeared in the SERS spectrum (Fig. 4 and Table S3†), which are consistent with the results in the literature.^11^ Compared to the Raman spectrum of CP, the trapping of guest molecules inside molecule cages with multiple interactions has a significant effect on the Raman spectra and thus results in the broadening effect.^9^ Probably due to the host–guest interaction, some peaks in the Raman spectra appear or disappear. In fact, a similar phenomenon was also observed in the literature.^12^ In the case of HQ, the characteristic peaks of the guest molecule, such as the out-of-plane deformation of the C–O bond at 403 cm^–1^,^11^ were also detected (Fig. 4 and Table S3†), indicating the capture of HQ in the cage. Moreover, CP and HQ were selectively detected with a limit of detection down to 10^–8^ and 10^–10^ M using the created hotspots as the SERS substrate (Fig. S30†). However, in contrast to both cases described above, due to the size or charge of guest molecules, no corresponding Raman signals were observed for both DCTP and PDA, even after prolonged immersion times of the substrates (Fig. S31†), which is in agreement with the host–guest behavior of the Pd_2_L_4_ cage found in the bulk solution. Importantly, we found that the trapped molecule inside the cage could be removed by washing with solvents at elevated temperature (60 °C) (Fig. S32†). All the results indicate that the embedded cages endowed the formed hotspots or substrates with the capability of selectively and reversibly trapping targeted molecules.

**Fig. 5:**
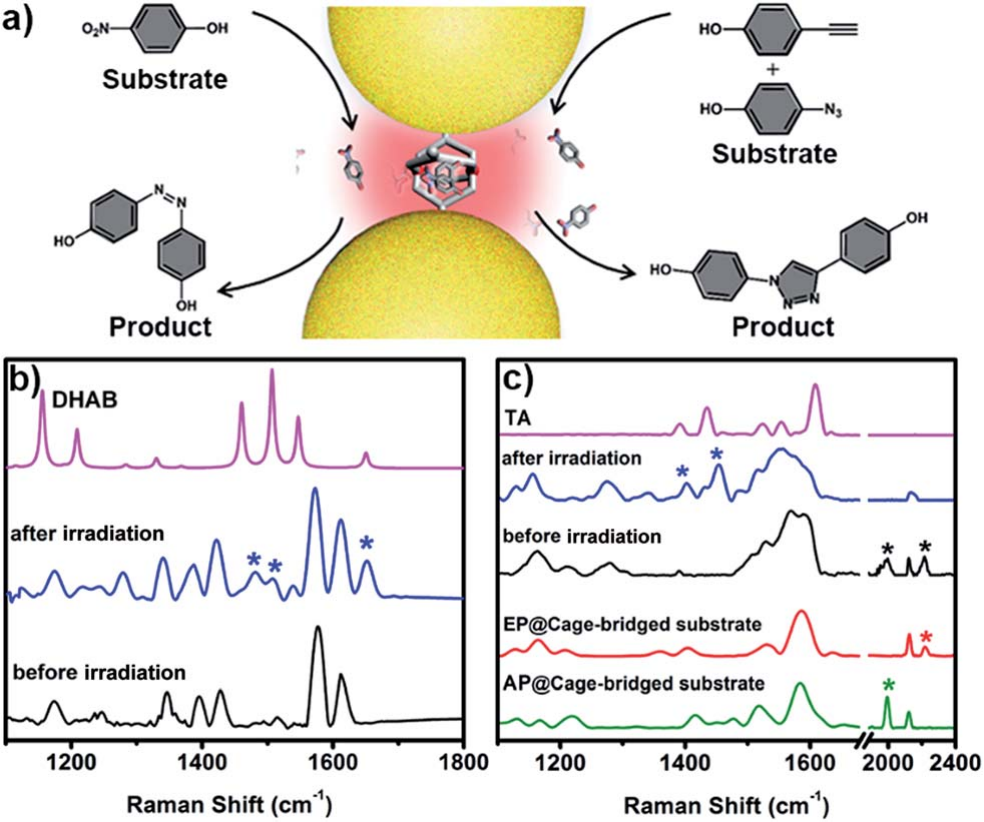
(a) Schematic illustration of the use of the created hotspots as a nanoreactor for plasmon-assisted chemical transformations; (b) SERS evidence of the formation of azobenzene through the photoreduction of 4-NP in the hotspots; (c) SERS evidence of the occurrence of the click reaction between 4-EP and 4-AP in the hotspots.

The unique capacity of plasmonic nanostructures to trap molecules that have a low affinity for metal surfaces and to selectively place the target molecules into hotspots could offer tremendous opportunities for numerous plasmon-assisted applications.^4^ As a demonstration, in this work, the plasmonic hotspots created using the Pd_2_L_4_ cage were utilized as a novel nanoreactor for facilitating chemical transformations by exploiting plasmon-induced heating and energetic charge-carriers (electron–hole pairs). 4-NP was firstly chosen as a substrate to be converted into the product dihydroxylazobenzene (DHAB), as shown in Fig. 5a. Fig. 5b shows the evolution of the performed chemical reaction inside the cage-bridged plasmonic clusters monitored using SERS. Initially, only the Raman signal of the captured 4-NP at 1346 cm^–1^ (–NO_2_ γ stretching) was detected. After the irradiation of plasmonic clusters under laser irradiation (1 mW) at 633 nm for a given time, three bands associated with DHAB at *ν*_1_ = 1481 cm^–1^, *ν*_2_ = 1509 cm^–1^ (–N=N–stretching) and *ν*_3_ = 1652 cm^–1^ (*ν*_CC_ stretching) appeared,^13^ which are consistent with the Raman spectrum of the commercially available DHAB compound. Besides the photoreduction reaction of 4-NP, it was interesting to find that the azide–alkyne Huisgen 1,3-dipolar cycloaddition could take place smoothly without the use of a catalyst. Generally, the activation of such so-called click reactions always needs a catalyst and a good deal of heating. In our case, probably due to the plasmon-induced heating in the hotspots as well as the spatial preorganization of the reactants in a confined cage, the cycloaddition reaction becomes possible under mild conditions. 4-Ethynylphenol (4-EP) and 4-azidophenol (4-AP) were used as starting compounds (Fig. 5c). As shown in Fig. 5c, after irradiation at 633 nm for a given time, two characteristic bands of the triazo ring stretching at *ν*_1_ = 1400 cm^–1^ and *ν*_2_ = 1453 cm^–1^ were detected,^14^ and the bands of azido and alkynyl of the starting compounds disappeared, which was indicative of the occurrence of the cycloaddition reaction. Certainly, as controlled experiments, the plasmonic clusters prepared by simple deposition of the same Au NPs on a glass substrate were used for the same reactions under identical conditions, but no corresponding reactions were observed (Fig. S33†). Blank experiments using a cage in the absence of gold nanoparticles were also performed. After encapsulating guest 4-NP in the cage, the sample was irradiated under laser irradiation (1 mW) at 633 nm for a given time. Fig. S34† shows the comparison of the Raman spectra of the host–guest complex before and after laser irradiation. Clearly, after irradiation the bands associated with –NO_2_ stretching remain unchanged and no bands associated with –N=N stretching appeared over time, indicating that no reaction occurred in the molecular cages in the absence of gold nanoparticles under identical conditions. Similar results were also observed for the case of the cycloaddition reaction (Fig. S35†). These results clearly indicate that the use of the Pd_2_L_4_ molecular cage not only can efficiently generate the highly desired plasmonic hotspots with small (1.2 nm) separation, but can also simultaneously impart the capability of trapping low affinity guests in the created hotspots, which could greatly expand the applications of plasmon-assisted chemistry. Ideally, the blank experiment of reactions will need to be performed with gold nanoparticles and cages that are not linked. In our system, however, due to the presence of thioether moieties at the periphery of the used cage, it is impossible or difficult to create an ideal system consisting of gold nanoparticles and cages that are not linked to perform the desired control experiments. Thus, in the future, we will perform a systematic study to address this issue through the synthesis of the same molecular cage without thioether or thiol groups.

## Conclusions

Creating well-defined plasmonic hotspots with enormous field enhancement as well as the capability of selectively trapping targeted molecules into hotspots is critical for numerous plasmon-assisted applications, but represents a great challenge. In this work, based on the assembly of Au NPs using a metal–organic molecular cage as a linker, a series of cage-bridged plasmonic nanostructures were developed. It was found that the integration of host–guest chemistry of the molecular cage with the plasmon coupling of Au NPs enables the formed plasmonic structures to address the mentioned challenges in terms of the formation of well-defined hotspots, the capture of molecules with lower affinities to the metal surface, the favourable molecule-accessibility, and the selective placement of target molecules in hotspots. The chosen applications above clearly highlight the significant value and tremendous potential of the cage-bridged plasmonic structures. Although one molecular cage was demonstrated in our case, the strategy described is generally applicable to other molecular cages for creating desired plasmonic nanostructures. As the described molecular cage is constructed through the coordination of organic linkers and metal ions, a careful selection of ligands and metal ions permits control of the geometric and host–guest characteristics of the formed cages in a broader range. Such attractive attributes imply that the precise design and tuning of hotspots in plasmonic structures and their molecule-trapping properties are possible. Thus, our work opens a new avenue for engineering plasmonic architectures and affords a new class of plasmonic nanostructure, which could offer huge opportunities for numerous plasmon-based applications.

## Author contributions

C. W. planned and performed the experiments, analysed the data and wrote the paper. L. T., N. G. and K. Z. assisted in the experimental work. S. W. assisted in the theoretical simulation. W. Z., X. Y., and W. Z. discussed the results. G. L. guided the project and wrote the paper.

## Conflicts of interest

There are no conflicts to declare.

## Supplementary Material

Supplementary information

Crystal structure data
