# Supplementary material for: Molecular cage-bridged plasmonic structures with well-defined nanogaps as well as the capability of reversible and selective guest trapping
Source: Chem Sci. 2017 Nov 20;9(4):889–95. doi: 10.1039/c7sc03536e (PMC5873215; doi:10.1039/c7sc03536e)
Supplement: Supplementary file 1 [file SC-009-C7SC03536E-s001.pdf]

Electronic Supplementary Material (ESI) for Chemical Science.
This journal is © The Royal Society of Chemistry 2017

Molecular cage-bridged plasmonic structures with well-defined nanogaps as well as the capability of reversible and selective guest trapping

Chen Wang, Li Tian, Wei Zhu, Shiqiang Wang, Ning Gao, Kang Zhou, Xianpeng Yin, Wanlin Zhang, Liang Zhao, and Guangtao Li*

Table of Contents

Table of Contents 2
Experimental Procedures 3
Chemicals. 3
Characterization. 3
Synthesis of organic ligand and cage 3
Single-Crystal X-ray Crystallography 4
Host-guest interaction 4
Results and Discussion 5
Guest Binding 6
UV Binding constant determination. 6
^{1}H NMR Titrations. 6
Preparation of plasmonic clusters 13
Near field and SERS Calculation 14
Preparation of Plasmonic Substrate 15
SERS Study 17
References 19

Experimental Procedures

Chemicals.

2,6-dibromoisonicotinic acid, sodium borohydride ($NaBH_4$), phosphorus tribromide (PBr_3), sodium methanethiolate ($NaSCH_3$), trimethylsilylacetylene, triethylamine, tetrakis(triphenylphosphine)palladium (0), were purchased from Alfa, tetra-n-butylammonium fluoride (TBAF), copper (I) iodide (CuI) and 3-iodopyridine was purchased from Adamas-beta and used as received. Potassium carbonate, methanol (MeOH), dichloromethane (CH_2Cl_2), absolute ethanol, and other solvents were purchased from Beijing Chemical Reagents Company and used without further purification. Ultrapure water was purchased from Wahaha Company and used in the preparation of aqueous solutions. Glass microscope slides were obtained from VWR.

Characterization.

^{1}H NMR spectra was obtained using a JEOLJNM-ECA300 at 400 MHz. XPS data was collected with a PHI5300 ESCA instrument using an Al K X-ray source. The UV/vis absorption spectra were recorded using a Perkin-Elmer UV/vis Lambda35 spectrometer. Scanning electron microscopy (SEM) images were obtained with a field-emission scanning electron microscope (LEO-1503, Germany). TEM image was obtained with a using JEM 2010 high-resolution transmission electronic microscope at an acceleration voltage of 100 kV. Raman spectra were measured upon excitation with a 633 nm laser line. Inelastically scattered light was collected with a LabRam HR system (Horiba-Jobin Yvon), equipped with a confocal optical microscope, high resolution gratings (1800 g mm^{-1}), a Peltier CCD detector and an x, y, z motorized stage. Spectra were collected by focusing the laser line onto the sample, using a 50× objective (N.A. 0.5), with accumulation times of 90 s and laser power at the sample of 1 mW.

Synthesis of organic ligand and cage

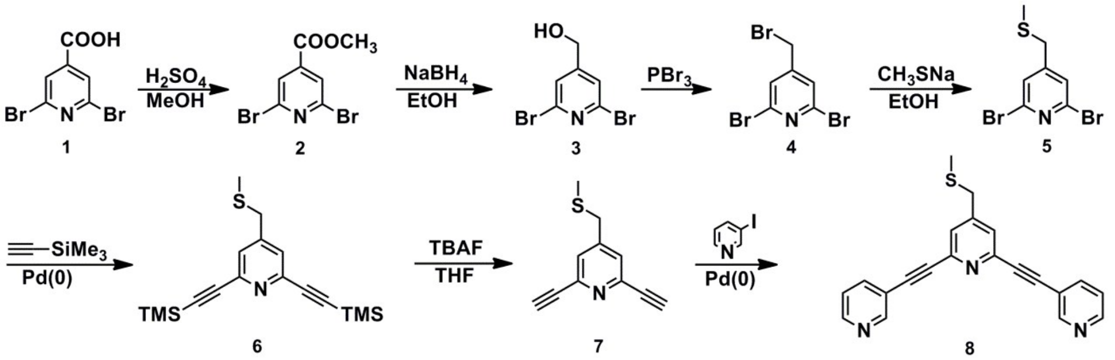

Scheme S1. Synthesis of 3,3'-((4-((methylthio)methyl)pyridine-2,6-diyl)bis(ethyne-2,1-diyl))dipyridine (L).

methyl 2,6-dibromoisonicotinate (2): A solution of **1** (31.0 g, 110.0 mmol) in MeOH (170 mL) was treated with conc. H_2SO_4 (1.4 mL) and refluxed for 16 h. After the mixture was cooled to room temperature, a white precipitate formed. The crude product was filtered and chromatographed on silica gel with CH_2Cl_2 to afford 25 g of **2** (75 %) as a white solid. ^{1}H NMR (400 MHz, $CDCl_3$): δ 3.97 (s, 3H), 7.99 (s, 2H).

(2,6-dibromopyridin-4-yl)methanol (3): Sodium borohydride (15.87 g) was added to a suspension of the **2** (25.0 g, 8.0 mmol) in 250 mL of ethanol. The mixture was refluxed for 3 h and cooled to room temperature. The ethanol was removed under vacuum, and then 320 mL of 1M HCl aqueous solution was added to decompose the excess sodium borohydride. The pH of the reaction mixture was adjusted to 7 by addition of saturated aqueous Na_2CO_3. The resulting solution was extracted with ethyl acetate (3 × 200 mL) and dried over anhydrous sodium sulfate, and the solvent was removed under vacuum. The desired solid was obtained in 53% yield and was used without further purification. ^{1}H NMR (400 MHz, $CDCl_3$): δ 4.72 (s, 2H), 7.47 (s, 2H).

2,6-dibromo-4-(bromomethyl)pyridine (4): A solution of **3** (12.0 g, 45.0 mmol) in anhydrous 1,4-dioxane (100 mL) was heated at 40℃ for 30 min, then phosphorus tribromide (15 mL, 55.0 mmol) was added. After further heating for 30 min, the reaction continued at room temperature for 15h. The mixture was pull into saturated aqueous solution of Na_2CO_3 and extracted with ethyl acetate, then dried over anhydrous sodium sulfate and the solvent was removed under vacuum. The desired solid was obtained in 97% yield and was used without further purification. ^{1}H NMR (400 MHz, $CDCl_3$): δ 4.28 (s, 2H), 7.47 (s, 2H).

2,6-dibromo-4-[(methylthio)methyl]pyridine (5): Solid $NaSCH_3$ (3.428 g, 49.0 mmol) was added to a solution of **4** (13.47 g, 40.8 mmol) in dry THF (100 mL) and the mixture was stirred under N_2 for 16 h. The solvent was removed under vacuum, and the pH of the

reaction mixture was adjusted to above 10 by addition of saturated aqueous $NaHCO_3$. The solution was extracted with Et_2O (3 × 80 mL) and dried over anhydrous sodium sulfate. The crude residue was purified by column chromatography on silica gel with petroleum ether/ethyl acetate (5%) as eluent to give pure **5** (3.1 g, 25 %) as yellow solid. ^1H NMR (400 MHz, $CDCl_3$): δ 2.02 (s, 3H), 3.55 (s, 2H), 7.47 (s, 2H).

2,6-bis(ethynyl)-4-[(methylthio)methyl]pyridine (7): Under nitrogen gas trimethylsilylacetylene (2.94 g, 30.0 mmol) was added to a solution of **5** (2.98 g, 10.0 mmol) in deoxygenated distilled triethylamine (35 mL) and THF (35mL) with tetrakis(triphenylphosphine)palladium (0) (590 mg, 0.50 mmol) and CuI (38.1 mg, 0.2 mmol) in a 100 mL Schlenk flask. After stirring for 1 h at room temperature, the reaction system was heated to 60℃, at which the reaction solution was further stirred for 12 hours. The reaction system was cooled to room temperature and the THF was removed under vacuum. The mixture was dissolved in H_2O and extracted with CH_2Cl_2 three times. The combined dichloromethane layer was washed with brine and dried over sodium sulfate. After evaporation of the CH_2Cl_2, the crude **6** was brown oil. A solution of crude **6** (1.6 g, 4.8 mmol) in THF (30 mL) was reacted with TBAF (2.0 g, 14.4 mmol) at room temperature for 1 h. After removing the solvent, water was added and then the system was extracted with CH_2Cl_2 three times. The solution was dried over sodium sulfate. After the solvent evaporated, the crude residue was purified by column chromatography on silica gel with petroleum ether/ethyl acetate (5%) as eluent to give pure **7** (450 mg, 45 %). ^1H NMR (400 MHz, $CDCl_3$): δ 1.99 (s, 3H), 3.58 (s, 2H), 7.47 (s, 2H).

3,3'-((4-((methylthio)methyl)pyridine-2,6-diyl)bis(ethyne-2,1-diyl))dipyridine (8): Under nitrogen gas, 3-iodopyridine (1.0 g, 4.84 mmol) was added to a solution of **7** (450 mg, 2.42 mmol) in deoxygenated distilled triethylamine (5 mL) with tetrakis(triphenylphosphine)palladium (0) (400 mg, 0.35 mmol) and CuI (30.0 mg, 0.16 mmol) at room temperature. After stirring at reflux temperature for 20 hours, the reaction system was cooled to room temperature, to which a saturated aqueous solution of ammonium chloride and CH_2Cl_2 were added. The organic layer was separated and the aqueous layer was further extracted twice with CH_2Cl_2. The combined CH_2Cl_2 layer was washed with brine and dried over sodium sulfate. After evaporation of dichloromethane, the crude residue was purified by column chromatography on silica gel with CH_2Cl_2/MeOH (2%) as eluent to give pure **8** (246 mg, 25 %). ^1H NMR (400 MHz, CD_3CN): δ 1.86 (s, 3H), 3.62 (s, 2H), 7.34 (m, 2H), 7.50 (d, 2H), 7.88 (d, 2H), 8.50 (d, 2H), 8.74 (d, 2H); ^{13}C NMR (75 MHz, CD_3CN): δ 14.96, 36.87, 86.44, 91.72, 119.72, 124.37, 128.11, 139.87, 143.97, 150.61, 150.92, 153.26. ESI-MS (+): m/ z (+) = 342.27 $[M+H]^+$.

Synthesis of Pd_2L_4 cage: To a stirring solution of $[Pd(CH_3CN)_4](BF_4)_2$ (11.1 mg, 0.025 mmol, 1 eq) in acetonitrile (dry, 1.5 mL) was added **L** (17 mg, 0.05 mmol, 2 eq.). The reaction mixture was heated at 50 °C for 30 minutes. The product was precipitated as a pale yellow solid by vapor diffusion of diethyl ether into the cooled reaction mixture.

Synthesis of Pd_2L_4 cage single crystals: Through vapor diffusion of diethyl ether into 1.5 mL acetonitrile solution of ligand **L** (17 mg, 0.05 mmol, 2 equiv.) and $[Pd(CH_3CN)_4](X)_2$ (11.1 mg, 0.025 mmol, 1 equiv.) at room temperature (X= BF_4^- or OTf^-), the colorless cage single crystal was achieved after three days with good yields of 79-93%. ^1H NMR (400 MHz, CD_3CN): δ 1.89 (s, 3H), 2.14 (s, 2H), 3.64 (s, 2H), 7.61 (m, 2H), 7.63 (d, 2H), 8.15 (d, 2H), 9.04 (d, 2H), 9.32 (d, 2H). ^{13}C NMR (75 MHz, CD_3CN): δ 15.04, 36.66, 83.50, 94.51, 123.97, 128.64, 129.43, 143.39, 144.41, 151.24, 151.50, 154.49. ESI-MS (+): m/ z (+) = 394.64 $[Pd_2L_4]^{4+}$.

Calculation of hydrodynamic radius from DOSY experiment:
The general expression of Stokes-Einstein's equation for the diffusion of spherical particles in solution is $D = k_BT/6\pi\eta r$
Where D = diffusion coefficient; k_B= Boltzmann's constant; ($k_B = 1.3806 \times 10^{-23}\ m^2kgs^{-2}K^{-1}$);
T = absolute temperature; η=dynamic viscosity of the solvent; r=hydrodynamic radius of a spherical particle.
Experimental condition
T= 298 K; η (MeCN-d_3 @ 298K) = $0.39*10^{-3}$ kg m^{-1} s^{-1} （20℃）
D= $4.9*10^{-10}$ m^2/s
d= 1.14 nm

Single-Crystal X-ray Crystallography

Single-crystal X-ray diffraction data were obtained on a D/max-RB (Japan, Rigaku) X-ray diffractometer equipped with a low temperature device and a fine-focus sealed-tube X-ray source (graphite monochromated Mo-Kα radiation, λ = 0.71073 Å, ω-scans with a 0.5^o step). Suitable single crystals were directly picked up from the mother liquor for data collections.

CCDC 1492105

Host-guest interaction

For the guest 4-NP and HQ, after adding guests into the CD_3CN solution of the cage, the obtained solution were examined by ^1H NMR, ESI-MS and UV-Vis. For the guest CP, DCTP and PDA, after adding guests into the CD_3CN solution of the cage, the obtained suspension was sonicated for 10 min, and then the solution of the mixtures were examined by ^1H NMR and ESI-MS. However, due to the limited solubility of CP, DCTP and PDA in CH_3CN solvent, the ^1H NMR titration and UV-Vis titration with the cages in CH_3CN can not be performed.

Results and Discussion

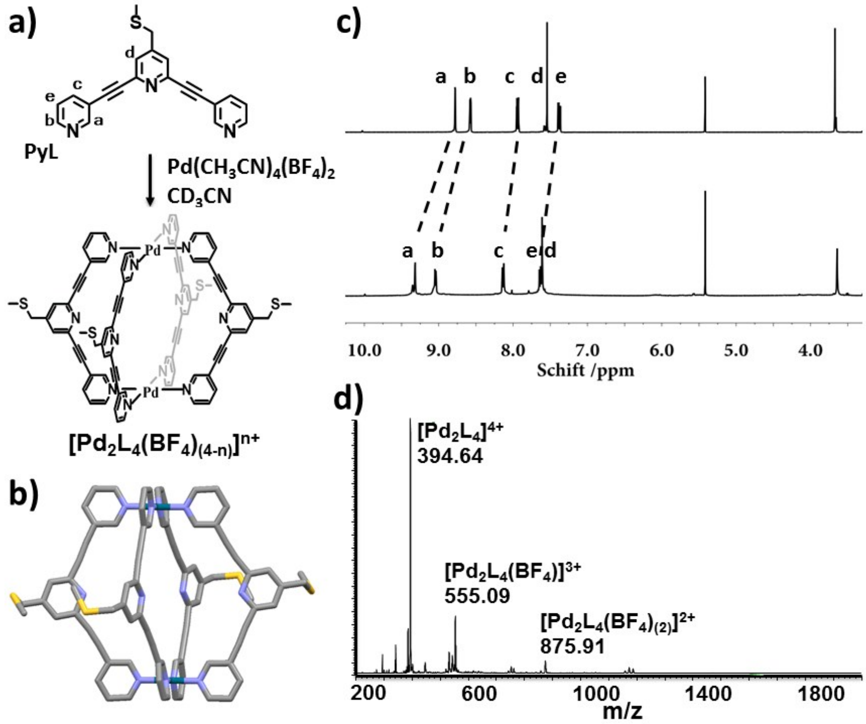

Figure S1. Synthesis and characterization of Pd_2L_4 cage that bear exo-thioether binging sites. a) Molecular structure of building block **L** and the assembly of cage; b) Crystal structure of cage; c) ^1H NMR spectra (298 K, CD_3CN) of building block **L** and cage; d) ESI-MS of cage with assignment of the observed species.

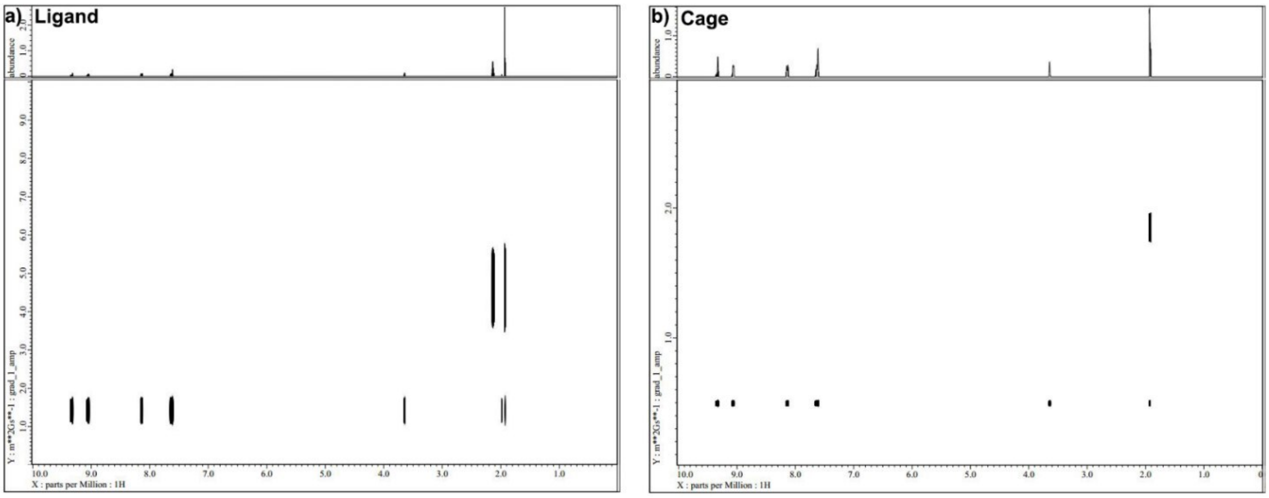

Figure S2. ^1H DOSY NMR spectrum (400 MHz, CD_3CN, 298 K) of **L** (a) and cage (b).

Table S1. Crystal data and structure refinements for cage

Compound cage			
Formula	$C_{88}H_{60}F_{12}N_{12}O_{12}Pd_2S_8$	b (Å)	15.243(5)
Calculated density	1.165 g/cm^3	c (Å)	17.550(7)
Fw	2172.8 g/mol	α (°)	100.64(4)
Crystal size (mm^3)	0.25×0.24×0.13	β (°)	102.20(5)
Color/Shape	Colorless block	γ (°)	98.20(6)
F(000)	1096	V (Å^3)	3099.8(18)
Crystal system	triclinic	Z	1
Space group	P-1	R (reflections)	0.0984
a (Å)	12.284(4)	wR_2 (reflections)	0.2873

Guest Binding

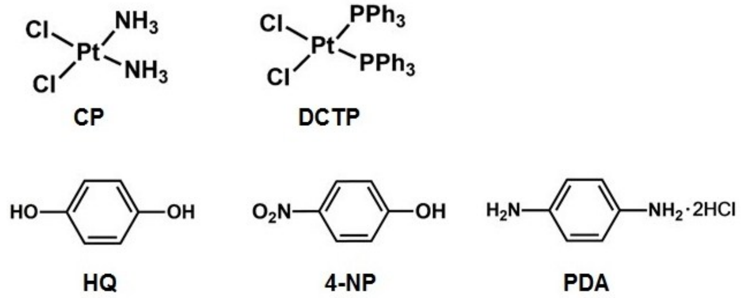

Scheme S2. The selected guest molecules with different size, shape and charge.

Table S2. The size of different guests [a]

	**a** (Å)	**b** (Å)	**c** (Å)
**CP**	**5.85**	**4.55**	**2.66**
**HQ**	**7.26**	**5.29**	**1.00**
**4-NP**	**7.53**	**5.16**	**2.11**
**DCTP**	**12.64**	**10.34**	**11.77**
**PDA**	**5.99**	**7.58**	**3.01**

[a] The size of guests is determined based on the optimized structures by using density-functional theory.

UV Binding constant determination.

Each titration study was conducted at least three times and the results show good reproducibility. Binding constants for 1:1 association were obtained according to the reported literatures.[1] Binding constants for 1:2 association were obtained according to the non-cooperative binding model using bindfit.[2]

^{1}H NMR Titrations.

For each titration, a solution of cage (1 mM) was titrated with a solution of guest (1 mM), maintaining a constant total concentration throughout. For each observable peak shift of cage in the ^{1}H NMR spectrum and the Job plot was applied to determine the binding.

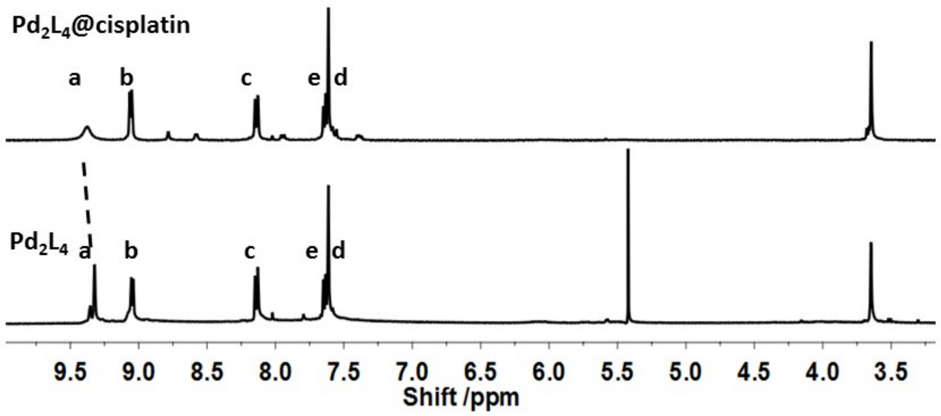

Figure S3. ^{1}H NMR spectra (298 K, CD_3CN) of Pd_2L_4 cage, and Pd_2L_4@cisplatin.

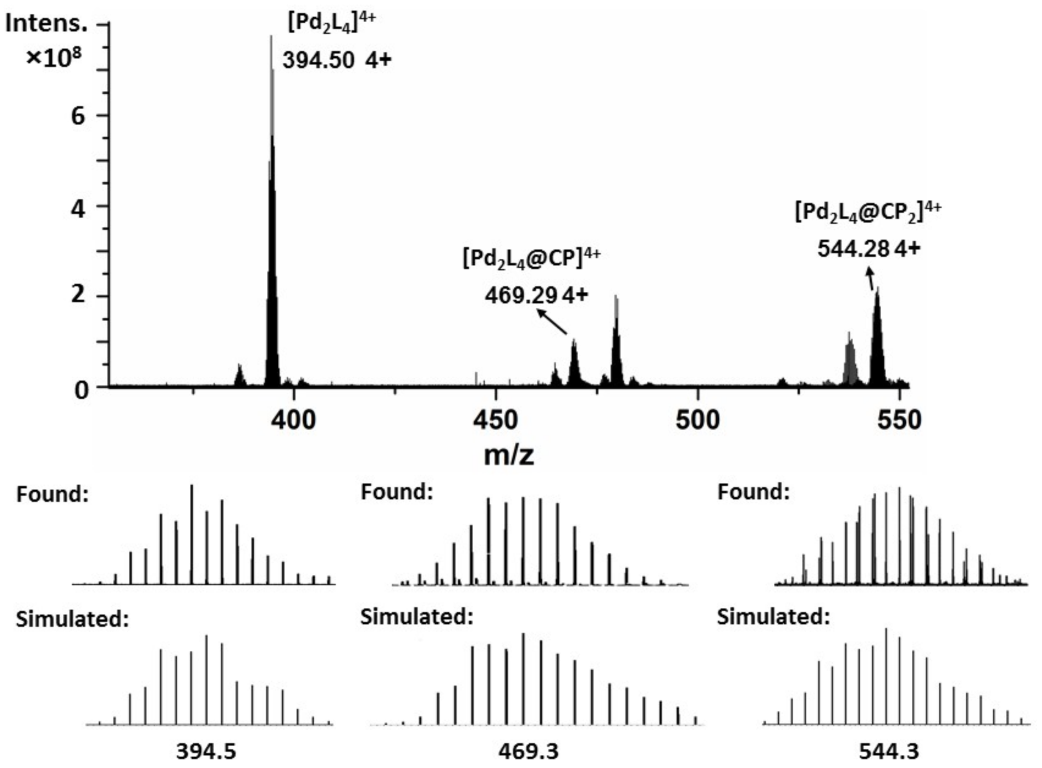

Figure S4. ESI-MS of $Pd_2L_4@CP_2$.

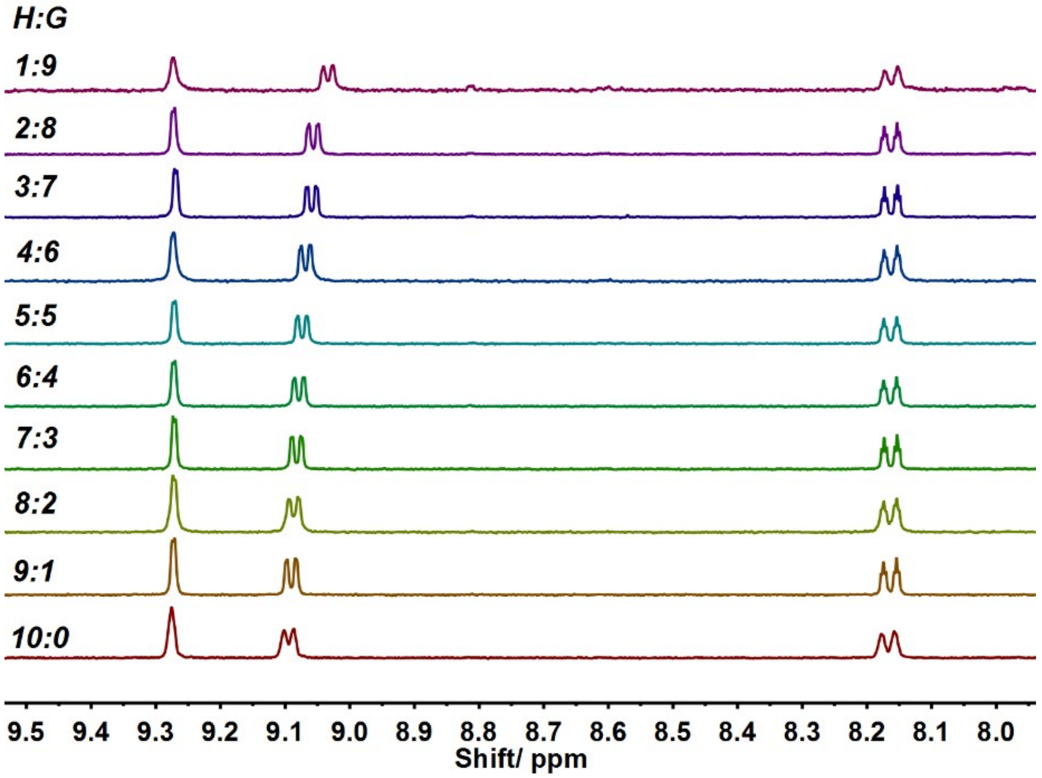

Figure S5. ^1H NMR titration (298 K, CD_3CN) of Pd_2L_4 with HQ.

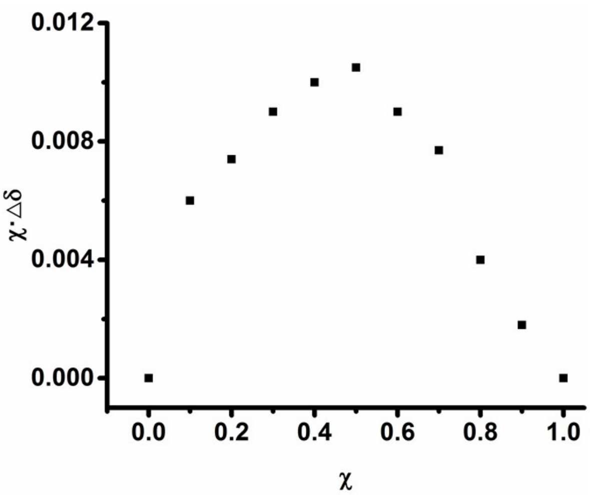

Figure S6. Job plot of Pd_2L_4 binding to HQ (298 K, CD_3CN). The experimental value of 0.5 indicate a H:G stoichiometry of 1:1.

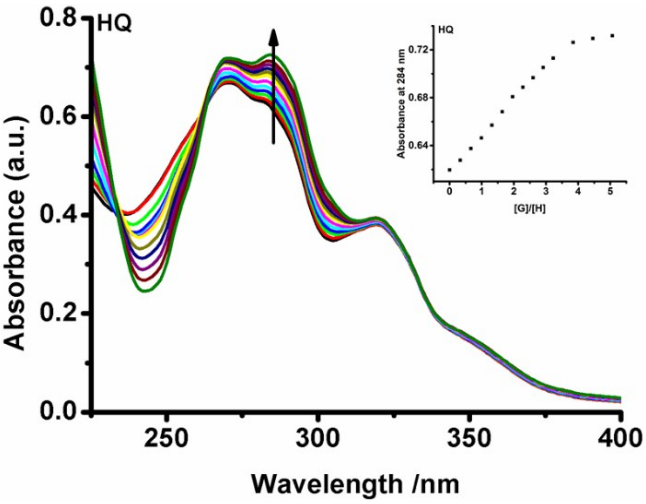

Figure S7. UV spectra of cage solution in CH_3CN (10^{-5} M) upon addition of HQ solution (10^{-3} M).

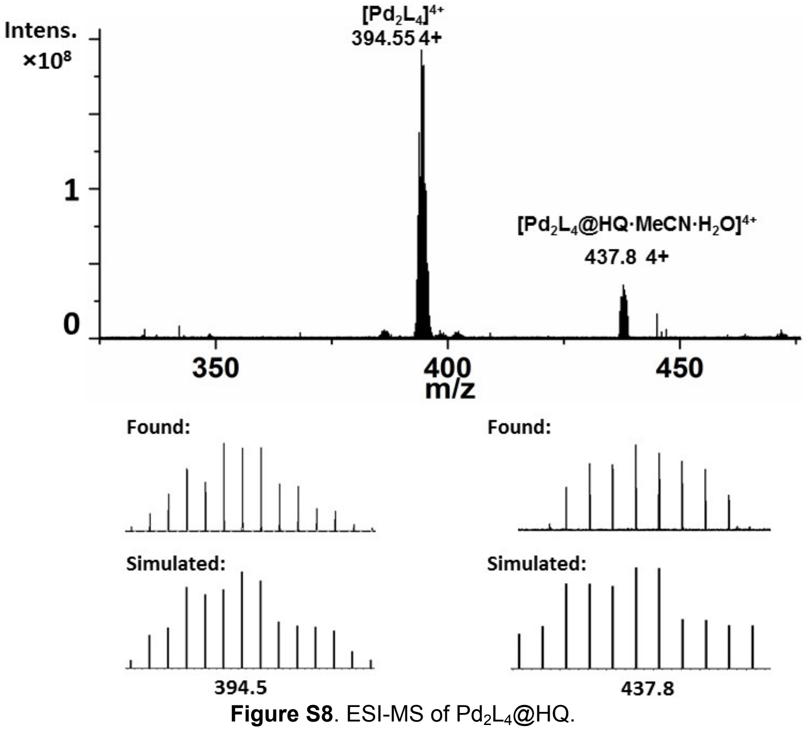

Figure S8. ESI-MS of Pd_2L_4@HQ.

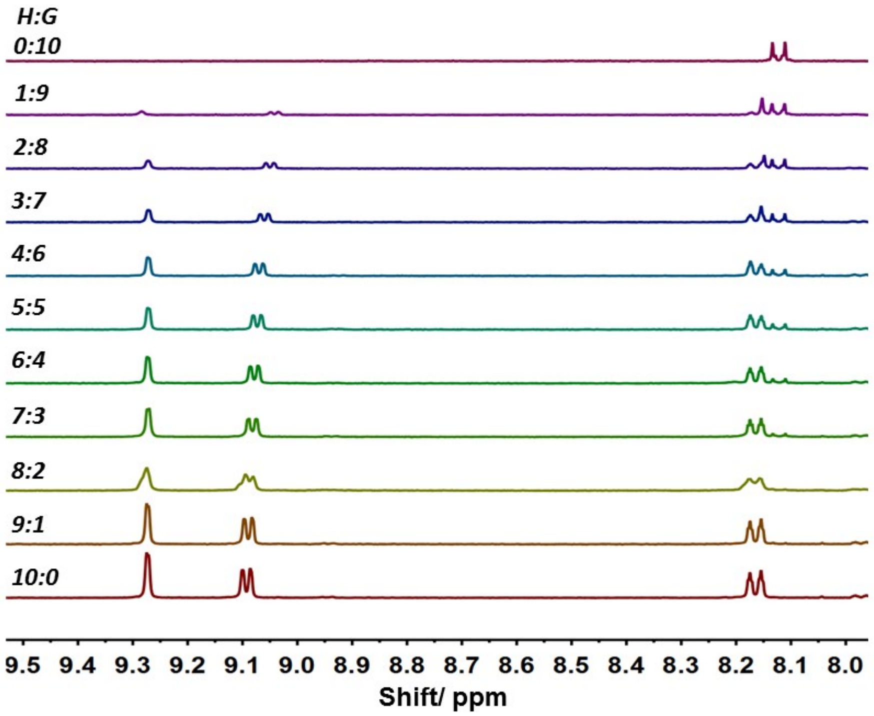

Figure S9. ^{1}H NMR titration (298 K, CD_3CN) of Pd_2L_4 with 4-NP.

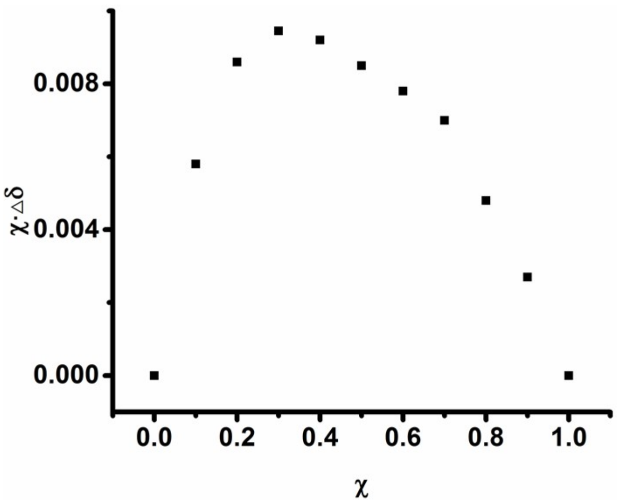

Figure S10. Job plot of Pd_2L_4 binding to 4-NP (298 K, CD_3CN). The experimental value of 0.3 indicate a H:G stoichiometry of 1:2.

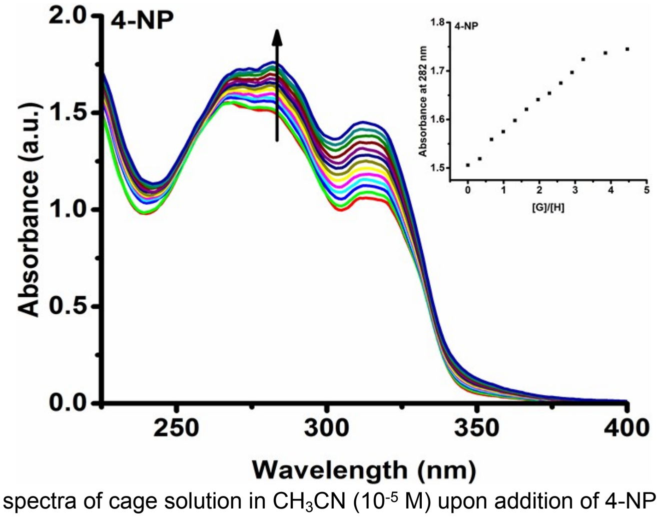

Figure S11. UV spectra of cage solution in CH_3CN (10^{-5} M) upon addition of 4-NP solution (10^{-3} M).

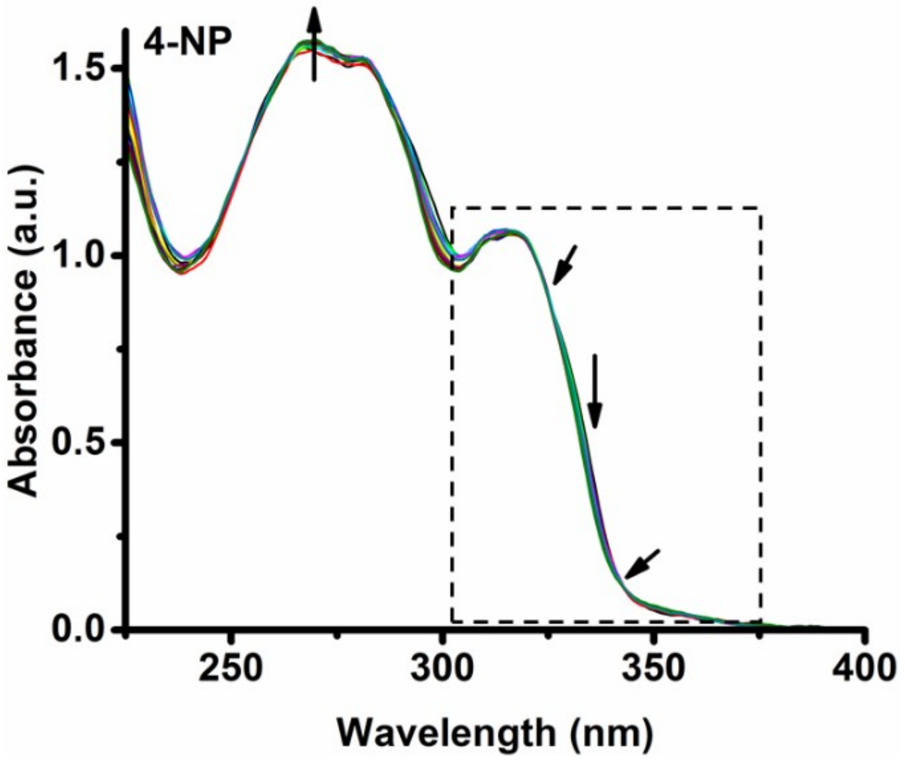

Figure S12. UV spectra of cage solution in CH_3CN (10^{-5} M) upon addition of 4-NP solution (10^{-3} M) deducting the contribution of the absorption of 4-NP.

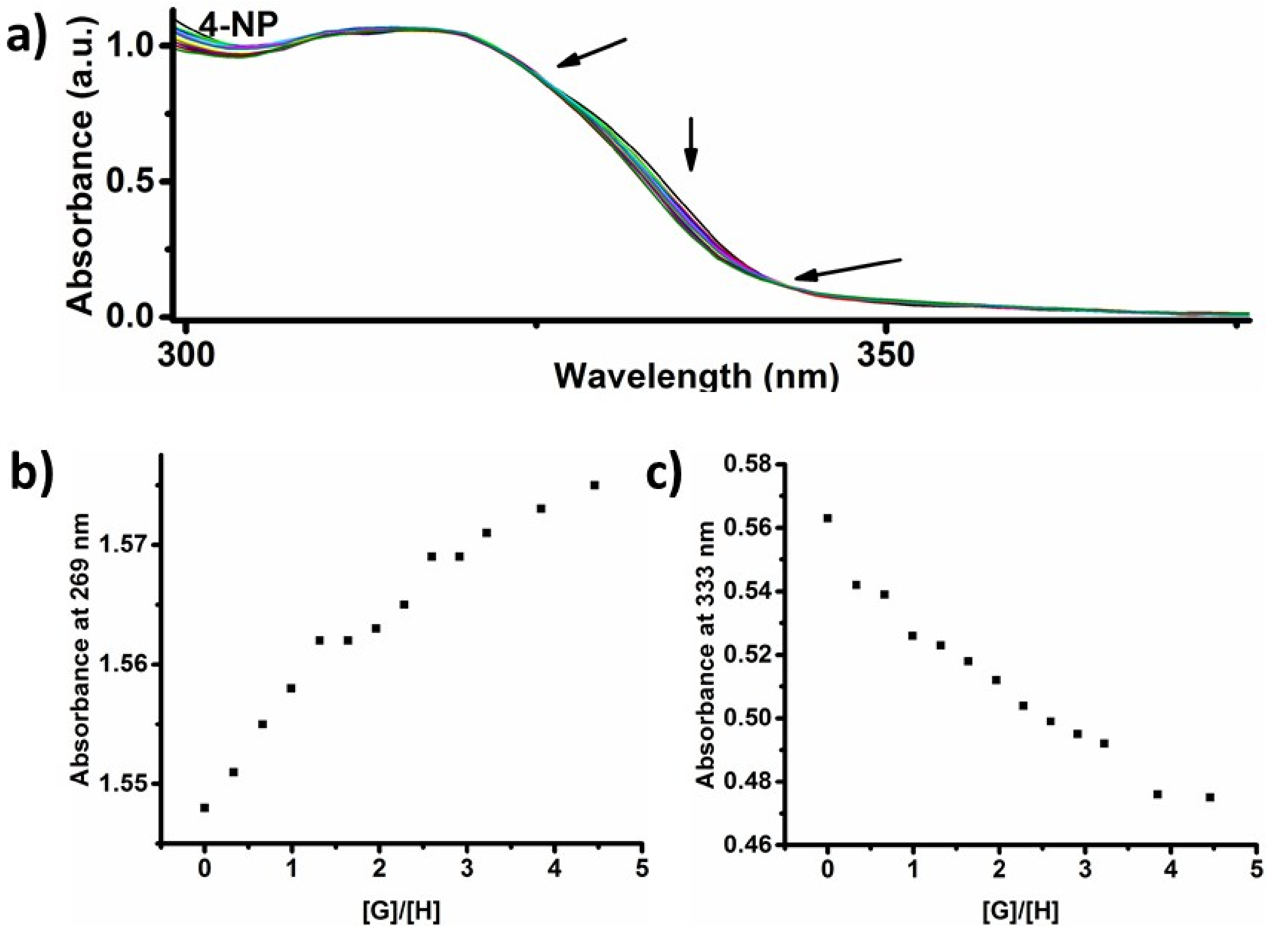

Figure S13. a) Enlarged UV spectra of cage solution in CH_3CN (10^{-5} M) upon addition of 4-NP solution (10^{-3} M) deducting the contribution of the absorption of 4-NP (K_1=4K_2, K_2 = 1.19×10^4 M^{-1}); b-c) the absorbance at 269 nm and at 333 nm.

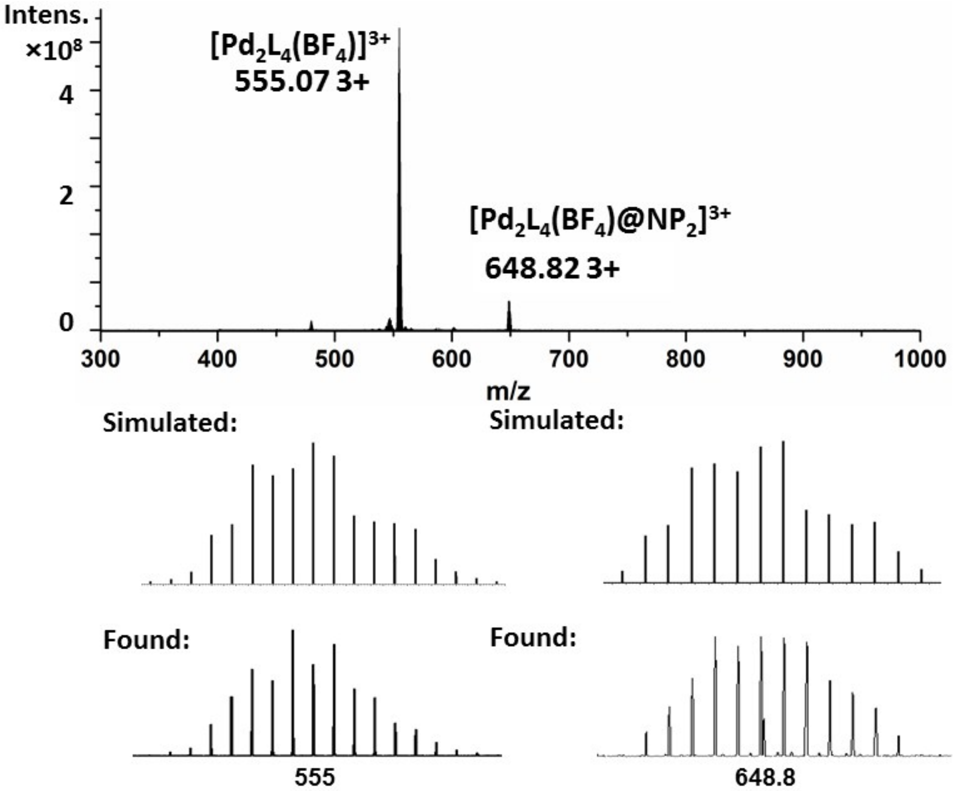

Figure S14. ESI-MS of Pd_2L_4@NP_2.

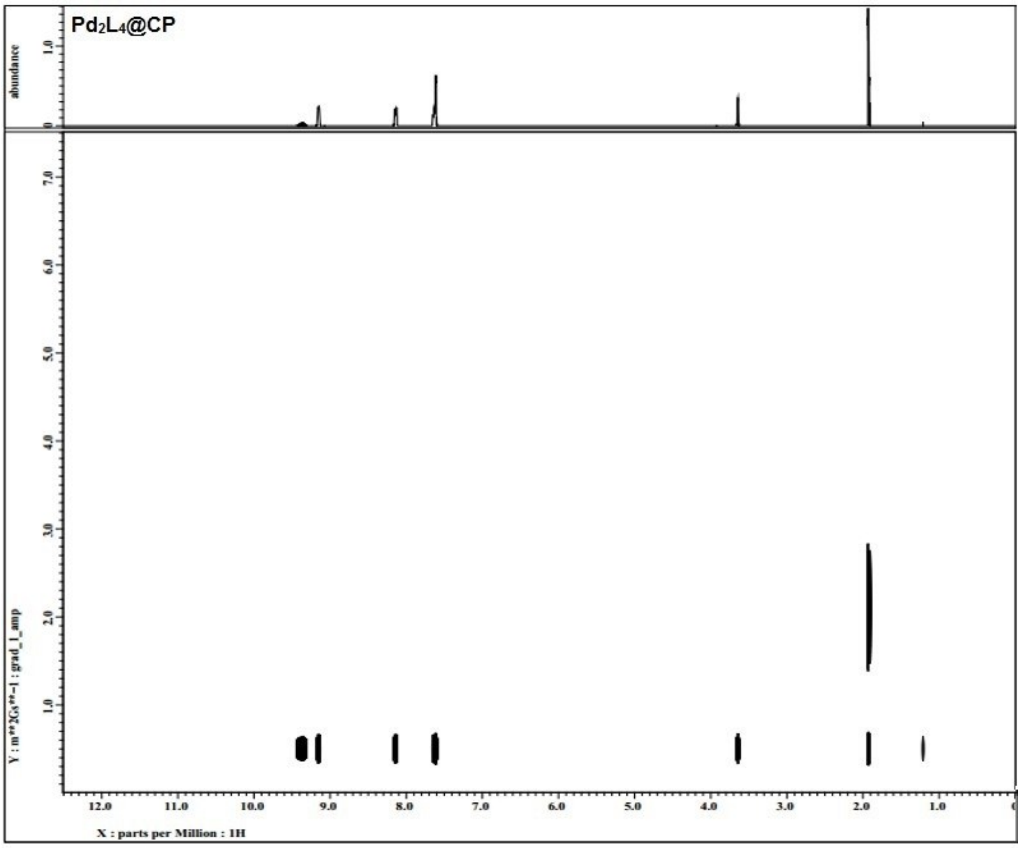

Figure S15. ^{1}H DOSY NMR of Pd_2L_4@CP.

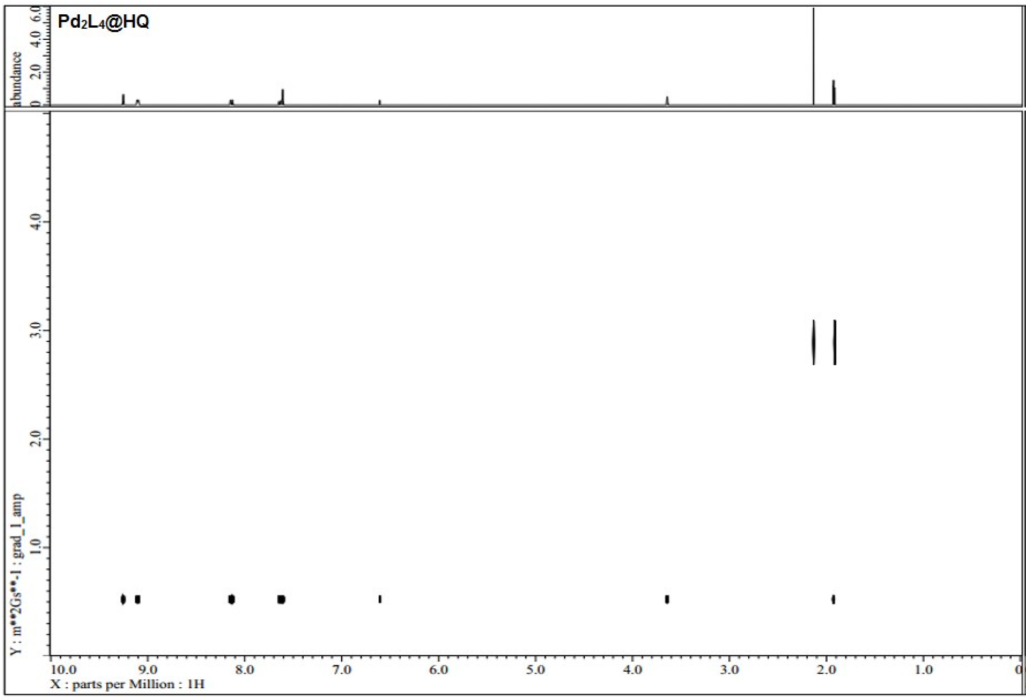

Figure S16. ^{1}H DOSY NMR of Pd_2L_4@ HQ.

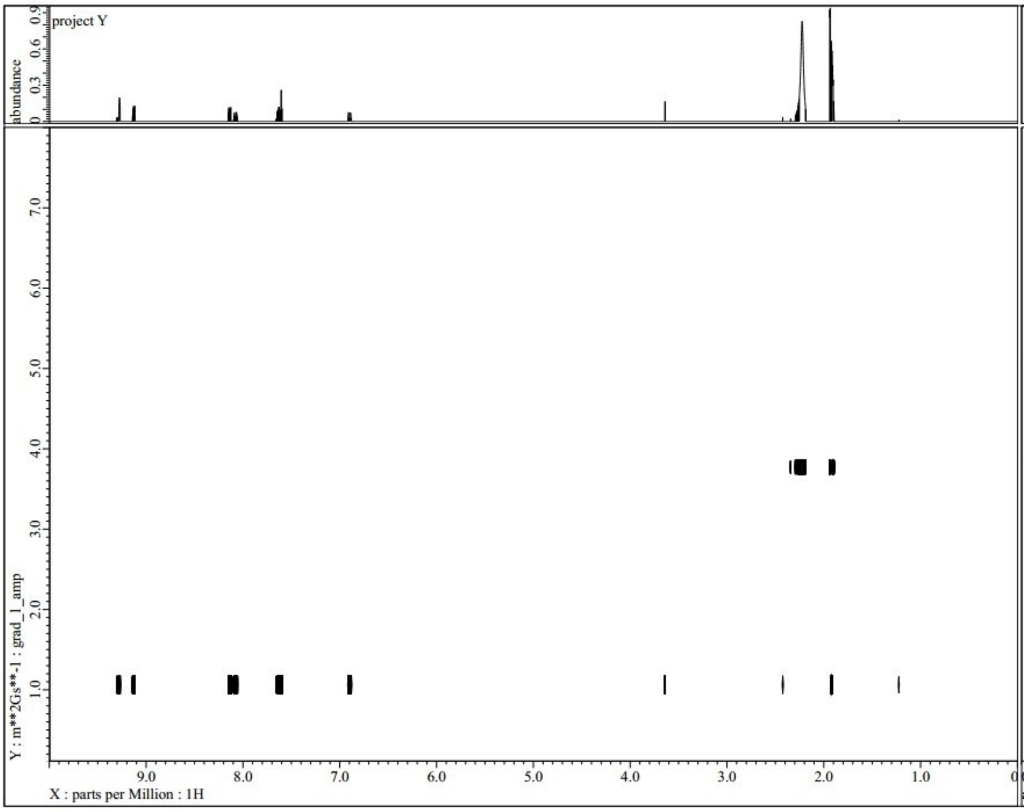

Figure S17. ^{1}H DOSY NMR of Pd_2L_4@ 4-NP.

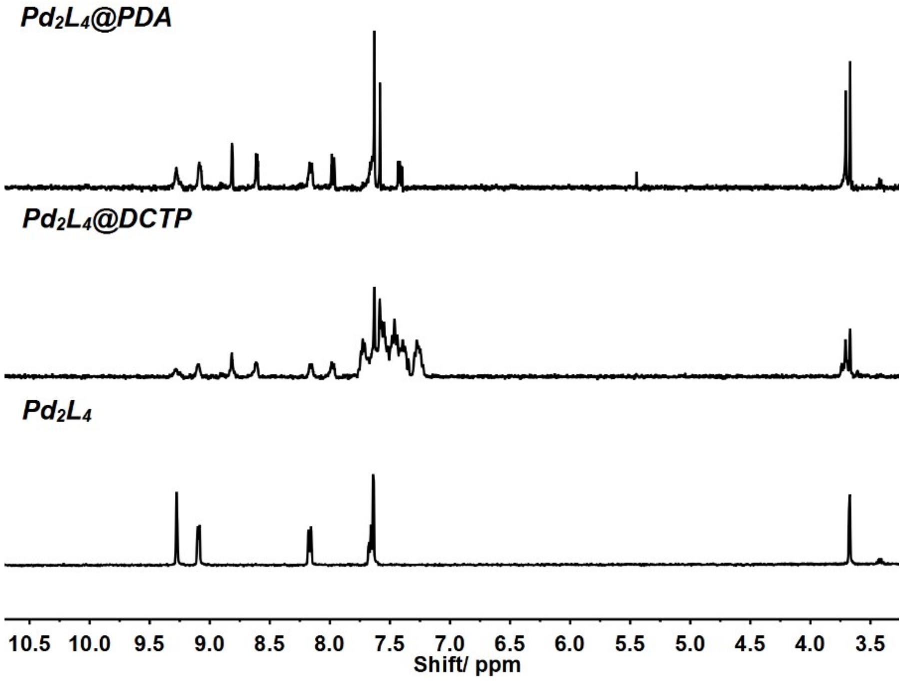

Figure S18. ^1H NMR spectra (298 K, CD_3CN) of Pd_2L_4 cage, Pd_2L_4@PDA, and Pd_2L_4@DCTP.

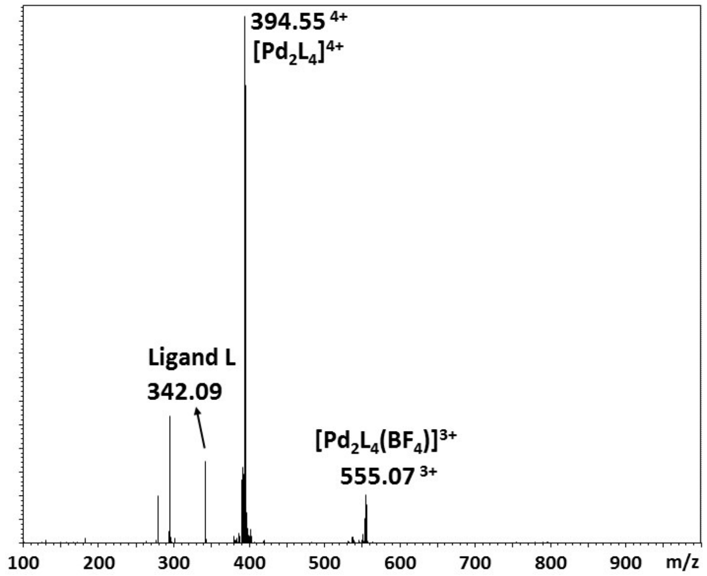

Figure S19. ESI-MS of Pd_2L_4@DCTP.

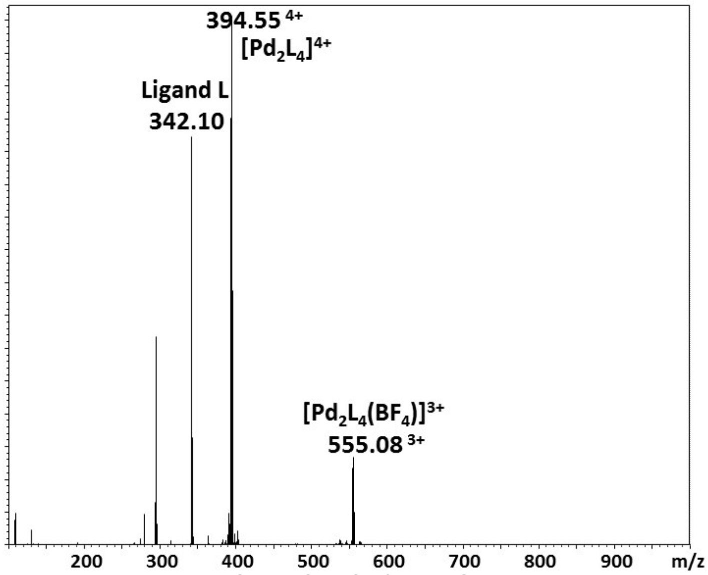

Figure S20. ESI-MS of Pd_2L_4@PDA.

Preparation of plasmonic clusters

The assembly of Au NPs with aid of the Pd_2L_4 cage in bulk gold colloidal solution.

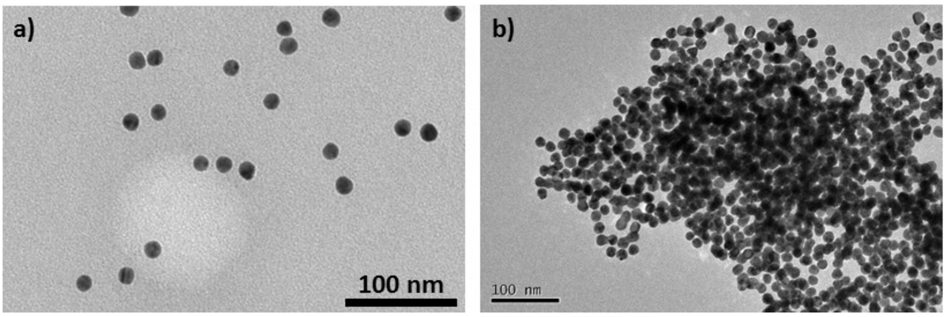

Figure S21. TEM images of original Au NPs (a) and huge aggregated Au NPs upon addition of the solution of Pd_2L_4 cage in bulk gold colloidal solution (b).

The assembly of Au NPs with aid of the Pd_2L_4 cage using microfluidic device.

Taking advantage of a microfluidic device to prepare the plasmonic clusters, the final concentration of cage and Au NPs was adjusted to be 10^{-7} M and 10^{-8} M, respectively. After reaction for 6 h, the different populations of clusters were purified by density gradient centrifugation. The density gradient was made by mixing different ratios of DMSO and glycerol (0-20% of glycerol by volume) and separation is then induced by the specific sedimentation velocity of each different cluster under centrifugation at 7500 rpm for 20 min. We found that a relative molar ratio of cage to the Au NP should be maintained between 50 and 100 to form the plasmonic structures with higher-CNs. When the molar ratio of cage was increased, a much faster reaction rate was observed and aggregates of NPs were formed.

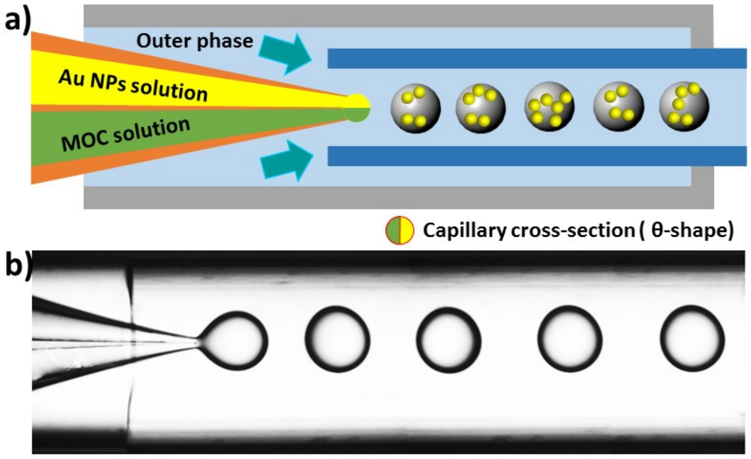

Figure S22. (a) Schematic illustration of the microfluidic device comprised of a tapered theta (θ)-shaped capillary for injection of two phases and a square capillary for droplet collection and preparation of plasmonic clusters; (b) Optical microscope image showing droplet generation.

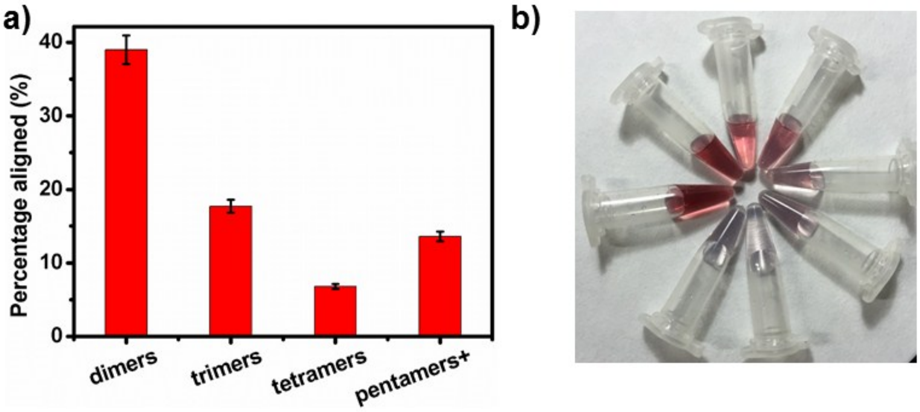

Figure S23. a) Histograms showing the percentage of clusters aligned; b) Optical microscope image of different clusters after density gradient centrifugation.

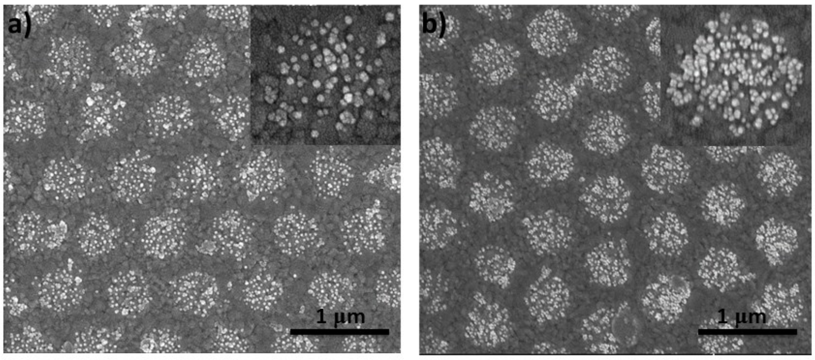

Figure S24. SEM images of regular arrays with varying CNs.

Near field and SERS Calculation

FDTD calculation. The FDTD method was employed to determine the electric field intensities and distributions at the surface of 15 nm Au NPs by FDTD Solution software. In the calculation, the incident electric field is defined as a plane wave with a wave vector that is normal to the injection surface. The overall simulation time was set to 1000 fs and calculated at the wavelength of 633 nm. For the UV-vis spectra, the refractive index is 1.4.

SERS EF calculation. Experimental EF was calculated according to the method proposed by Van Duyne et al.[3] Briefly, the following expression was used:

$$EF = \frac{I_{SERS} \times N_{normal}}{I_{normal} \times N_{SERS}}$$

where I_{SERS} is the surface-enhanced Raman intensity, N_{SERS} is the number of molecules bound to the enhancing plasmonic substrate, I_{normal} is the regular Raman intensity collected from solid molecules, and N_{normal} is the number of molecules in the excitation area.

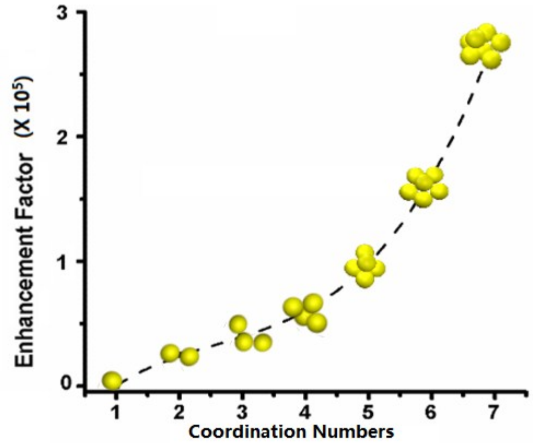

Figure S25. SERS enhancement factor of plasmonic clusters with different CNs.

Preparation of Plasmonic Substrate

The plasmonic substrate was prepared by layer-by-layer method. First, the substrate was modified by APS, followed by modification of Au NPs through the interaction between Au NPs and amino group of APS. Then through sequent immersion in the solution of Au NPs for 4 h and solution of cage for 2 h. The concentration of cage is 10^{-4} M

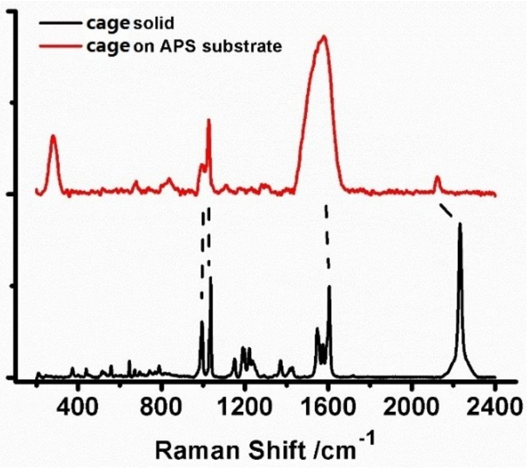

Figure S26. Raman spectra of cage solid and cage on plasmonic substrate.

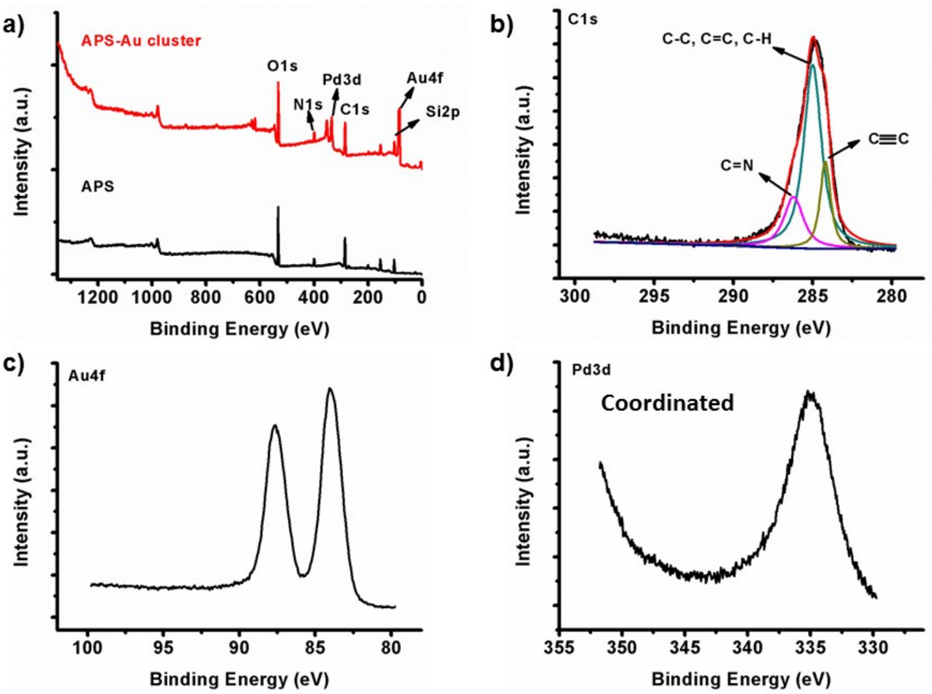

Figure S27. a) XPS spectra of APS monolayer and clusters on APS monolayer; b) high-resolution C1s spectra of the clusters on APS monolayer; c) high-resolution Au4f spectrum of the clusters; d) high-resolution Pd3d spectrum of the clusters.

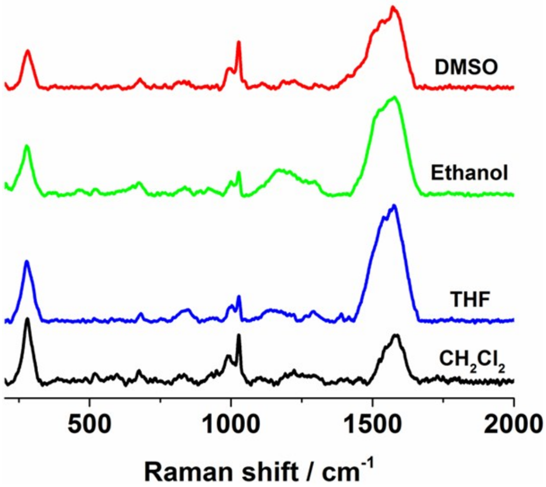

Figure S28. The SERS spectra of the cage-bridged Au clusters after treatment by different media.

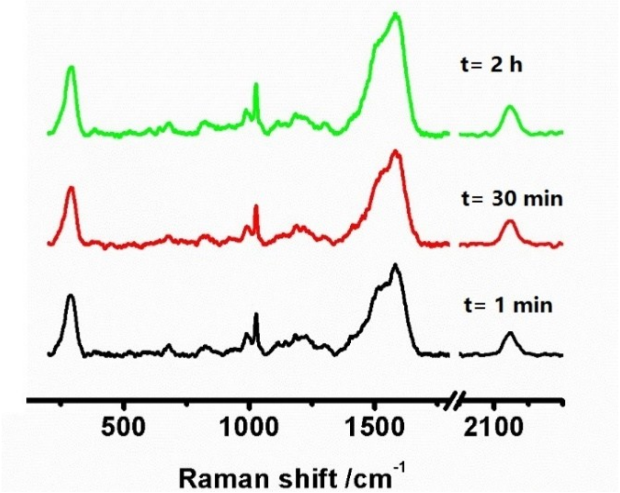

Figure S29. The SERS spectra of the cage-bridged Au clusters after continuous illumination for different periods.

SERS Study

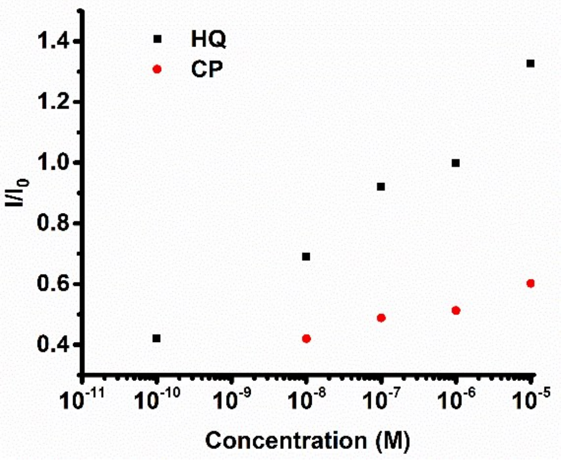

Figure S30. The Raman enhancement ratio (I/I_0) versus -[Analyte], and I represent the Raman intensities of CP at 501 cm^{-1} and HQ at 481 cm^{-1}, I_0 represent the Raman intensities of cage at 2187 cm^{-1}.

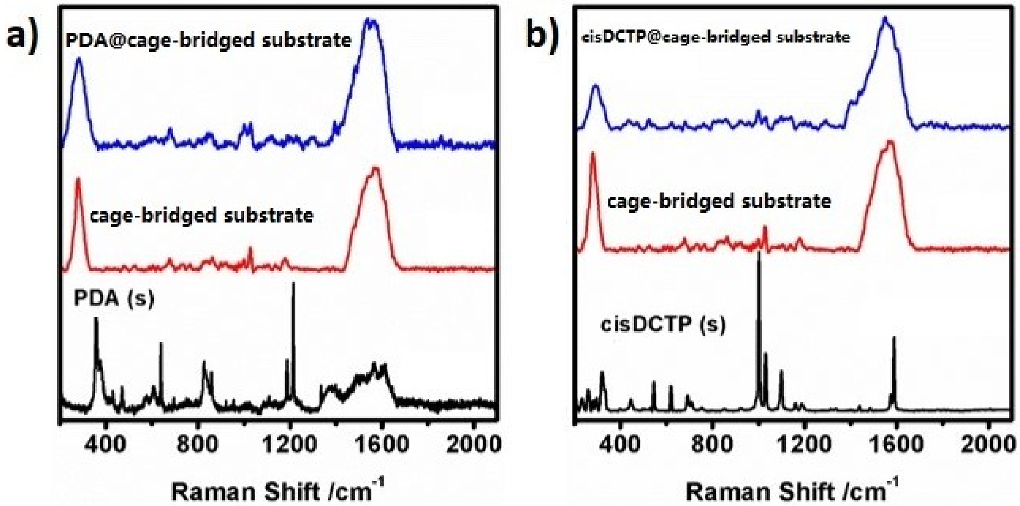

Figure S31. SERS spectra of trapping PDA and DCTP on the substrate.

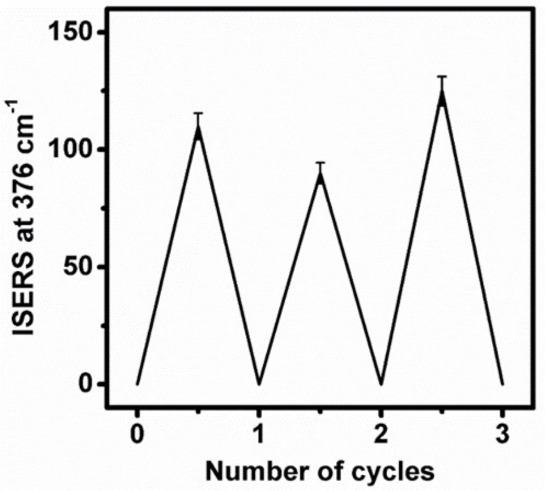

Figure S32. Cycling SERS activity.

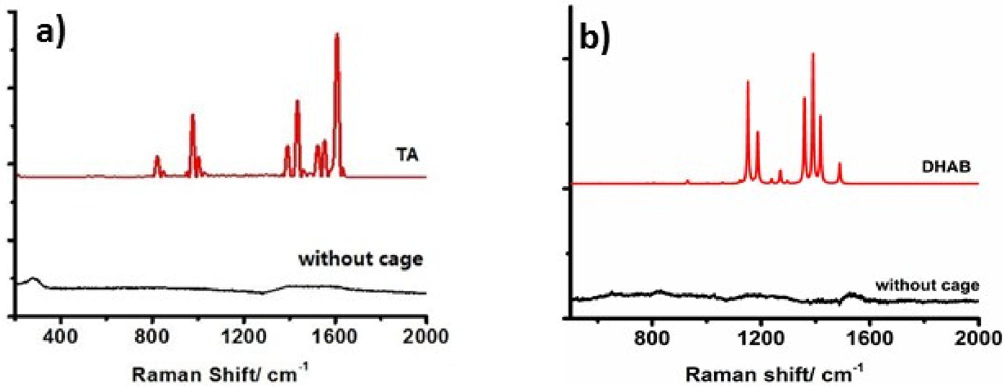

Figure S33. a) Raman spectra of the solid-state TA and the substrate without cages used for hot-spot reaction; b) Raman spectra of the solid-state DHAB and the substrate without cages used for hot-spot reaction.

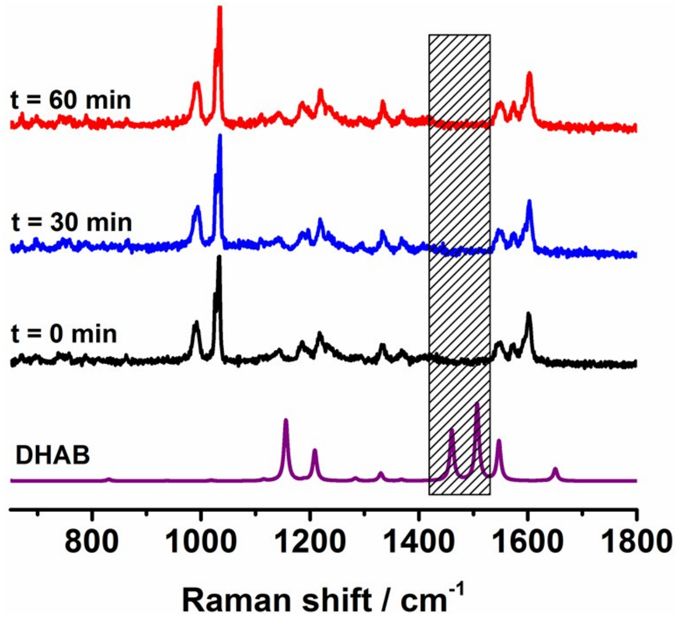

Figure S34. Raman spectra of the photoreduction of 4-NP in the absence gold nanoparticles under identical experimental conditions.

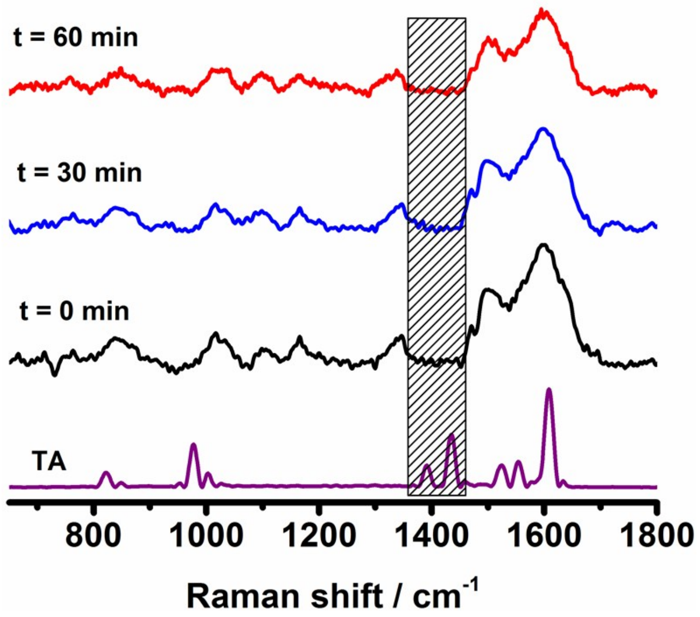

Figure S35. Raman spectra of the click reaction in the absence gold nanoparticles under identical experimental conditions.

Table S3. Raman peaks of guest molecules, cage and products

	trapped in clusters	in bulk (solid)	mode
CP	483	508	asymmetric and symmetric stretching
	501	524	doublet vibrations v(Pt-NH_3)
HQ	403	380	out-of-plane deformation of C-O bond
	481	477	CCO in plane bending
	663	645	CC stretching
Cage	999	996	ring-breathing vibrations
	1020	1027	ring-breathing vibrations
	1590	1609	C=C aromatic stretching
	2187	2232	alkynyl vibration modes
	1166	1152	C-H out of plane and in plane vibration
DHAB	1481	1460	-N=N-stretching
	1509	1506	-N=N-stretching
	1652	1650	v_{CC} stretching
TA	1400	1391	two characteristic bands of triazo ring
	1453	1435	stretching

References

[1] J. Bourson, J. Pouget, .B. Valeur, *J. Phys. Chem*. **1993**, *97*, 4552-4557.

[2] (a) Pall Thordarson, *Chem. Soc. Rev*. **2011**, *40*, 1305-1323; (b) Pall Thordarson, *Chem. Commun*., **2016**, *52*, 12792-12806.

[3] K. L. Wustholz, A. Henry, J. M. McMahon, R. G. Freeman, N. Valley, M. E. Piotti, M. J. Natan, G. C. Schatz, R. P. Van Duyne, *J. Am. Chem. Soc*. **2010**, *132*, 10903-10910.
